# Hybrid plasmonic nanodiamonds for thermometry and local photothermal therapy of melanoma: a comparative study

**DOI:** 10.1515/nanoph-2024-0285

**Published:** 2024-08-28

**Authors:** Elena N. Gerasimova, Landysh I. Fatkhutdinova, Ivan I. Vazhenin, Egor I. Uvarov, Elizaveta Vysotina, Lidia Mikhailova, Polina A. Lazareva, Dmitry Kostyushev, Maxim Abakumov, Alessandro Parodi, Vitaly V. Yaroshenko, Dmitry A. Zuev, Mikhail V. Zyuzin

**Affiliations:** School of Physics and Engineering, 65071 ITMO University, Lomonosova 9, 191002 St. Petersburg, Russia; Department of Medical Nanobiotechnology, N.I. Pirogov Russian National Research Medical University, Ostrovityanova 1 bldg. 6, 117997 Moscow, Russia; Martsinovsky Institute of Medical Parasitology, Tropical and Vector-Borne Diseases, Sechenov University, Moscow, Russia; Division of Biotechnology, Scientific Center for Genetics and Life Sciences, Sirius University of Science and Technology, Sochi, Russia; Faculty of Bioengineering and Bioinformatics, Lomonosov Moscow State University, Moscow, Russia; Laboratory of Biomedical Nanomaterials, National University of Science and Technology (MISIS), Leninskiy Prospekt 4, 119049 Moscow, Russia; Sirius University of Science and Technology, Olympic Ave, 1, 354340 Nizhneimeretinskaya Bukhta, Krasnodarskiy Kray, Sochi, Russia

**Keywords:** nanodiamonds, plasmonic nanoparticles, thermometry, photothermal therapy, melanoma

## Abstract

Hyperthermia plays a significant role in cancer treatment by inducing cell damage through temperature elevation, often used alongside other treatment modalities. During hyperthermia therapy, temperature control is crucial. Here, we report on a simple synthesis route of hybrid plasmonic nanodiamonds either completely wrapped with an Au shell (**NV@Au**) or densely covered with Au NPs (**NV@SiO**_**2**_ **@Au**). Such integration of nanodiamonds with Au NPs is advantageous both for heating and precise thermometry at nanoscale. After structural and optical investigations, heating abilities of the obtained plasmonic nanodiamonds were thoroughly inspected on glass, in association with living cells, and in tissue slices *ex vivo*, revealing their effective heat generation under excitation with light using a single excitation source. The developed hybrid plasmonic nanodiamonds were finally applied for local photothermal therapy of melanoma *in vivo*, demonstrating their efficacy in eradicating cancer cells and monitoring temperature during the process.

## 1 Introduction

The advantageous properties of heat have been recognized for centuries [1], [2]. For instance, mammals have evolved mechanisms to elevate their body temperature by 1–4 °C during inflammation, assisting in the resolution of certain pathological conditions [3]. In the field of oncothermia, elevated temperatures (hyperthermia) induce cell damage primarily through protein denaturation, offering a promising adjunctive therapy for tumor treatment, often in combination with other cancer treatment modalities [4]. Advances in materials science have enabled the development of methods to induce hyperthermia in tumor cells and tissues using nanoscale thermal agents that generate heat under external stimuli [5]–[8]. Notably, in the area of light stimulation, various nanomaterials were explored, such as plasmonic nanoparticles (NPs), capable of efficiently converting light energy into heat for applications in photothermal therapy (PTT) [9], [10]. Optically induced hyperthermia typically results in cell death through different mechanisms, including apoptosis at temperatures below 45 °C and necrosis at temperatures above 48–50 °C [11]. Clinical hyperthermia treatments typically distinguish between moderate-temperature hyperthermia (40–45 °C for 15–60 min) and high-temperature hyperthermia (above 50 °C for 4–6 min) [12]. Moreover, temperature elevation during hyperthermia impacts gene expression, metabolism, growth factor activity, cell division, and protein function, which makes temperature control during optical hyperthermia essential [13], [14].

Currently, several nanoscale temperature sensing techniques exist, each with its own advantages and limitations. These methods predominantly include Raman spectroscopy [5], [15], scanning probe microscopy [16], [17], and optical-based approaches, with the latter being particularly intriguing for their capability of real-time 3D temperature monitoring [14]. However, inaccuracies in temperature measurements using fluorescence probes (e.g., quantum dots) and issues such as irreversible decrease of fluorescence intensity, photobleaching, and photoblinking make optical-based thermometry challenging, especially in biological environments [18]–[20].

Fluorescent nanodiamonds with nitrogen-vacancy (NV) centers offer an exceptional alternative, emerging as one of the most promising materials for nanothermometry of biological objects due to their optical stability, biocompatibility, and resistance to photobleaching [21], [22]. In their electronic ground state, NV centers are a spin-1 system that can be manipulated with a microwave field and offer easy optical detection [23]–[25]. When the microwave frequency matches the frequency of the electron paramagnetic resonance transition of NV centers, a spin excitation from *m*_*S*_ = 0 to *m*_*S*_ = ±1 occurs, leading to a decrease in fluorescence intensity: this phenomenon is known as optically detected magnetic resonance (ODMR). The frequency of ODMR is temperature-dependent, enabling high-precision temperature measurements [26]–[28]. Thus, leveraging NV centers will provide meticulous temperature monitoring at the nanoscale, allowing prevention of overheating of biological objects. Notably, reported temperature resolution of ODMR-based thermometry technique depends on the used setup and lies in sub-millikelvin range for MW modulation scheme for cw-ODMR [29]. Furthermore, NV centers also provide temperature measurements with high resolution due to the coherent quantum properties that make nanodiamonds independent from medium conditions and calibration. The quantum constant d/d*T* = 2*π* × 74.3 kHz K^−1^ remains the same for a broad temperature range, which allows avoiding a nonlinear translation of the ODMR peak into temperature [27], [30].

However, to imbue NV centers with the additional property of optical heating, they must be combined with other elements, such as plasmonic nanoparticles [27]. On their own, NV centers cannot generate heat when exposed to light. Integrating NV centers with plasmonic nanostructures is challenging, although there are various methods for achieving this synergy. One of these methods employs supportive materials such as calcium carbonate particles [31]–[35], polymeric carriers [36], [37], or silica particles [38], [39]. While this approach stabilizes both plasmonic nanoparticles and NV centers, it cannot provide their precise spatial localization on the supportive materials, thus limiting repeatability of the optical properties. An alternative approach involves direct deposition of gold (Au) NPs onto the surface of NV centers [40], [41]. This method enables precise control over the minimal distance between the two entities, ensuring excellent temperature sensitivity. Additionally, the spatial arrangement of Au NPs allows for the customization of electromagnetic near-fields, creating localized hotspots crucial for enhancing nonlinear harmonic generation, surface-enhanced Raman spectroscopy, and other nonlinear optical effects [42]–[44]. Although up to now, few attempts have been made to deposit Au NPs onto the surface of nanodiamonds, the potential benefits warrant further exploration. Recently, it has been reported that nanodiamonds can be modified with Au via bottom-up DNA self-assembly. This approach allows the fabrication of various predictable morphologies of nanoassembly using two complementary ssDNA sequences, but it requires multistage preparation of Au and nanodiamonds surfaces. Another approach is more straightforward and involves the absorption of Au NPs on nanodiamonds’ surface by mixing them together [45], [46]. For this, pristine nanodiamonds were preliminarily functionalized with carboxyl groups: covered with poly-L-arginine hydrochloride and then shaken together with CTAB-modified Au nanorods to create hybrid nanostructures. Nonetheless, this modification technique suffers from unpredictable and uncontrollable nanohybrid morphology, because the amount of attached Au nanorods can vary from one nanohybrid to another. Moreover, in this case, optical heating of Au nanorods and NV-center excitation are provided by two different lasers, which makes the experimental setup complicated. Therefore, there is an urgent need for an easy and repeatable approach for the preparation of hybrid nanodiamond-Au structures capable of precise thermometry inside biological objects and effective heating.

Considering all the aforementioned factors, we propose straightforward and low cost methods for the preparation of hybrid plasmonic nanodiamonds with different geometries. By developing new and modifying existing synthesis approaches, we succeeded in obtaining hybrid plasmonic nanodiamonds with various coatings: (i) nanodiamonds completely enveloped in an Au shell (**NV@Au**), and (ii) nanodiamonds adorned with Au NPs (**NV@SiO**_**2**_ **@Au**), alongside with (iii) **NV@SiO**_**2**_ and (iv) **NV**, serving as controls. Following a thorough structural and optical analysis of the obtained hybrid nanomaterials, their thermal properties were extensively evaluated outside cells, within *in vitro* cell cultures, and in *ex vivo* laboratory animal models for the first time. Notably, the developed plasmonic nanodiamonds require the same laser for optical heating and thermometry measurements. Our plasmonic nanodiamonds with optimal geometry provided effective heat generation in living cells and tissues under excitation using a single excitation source for inducing cell hyperthermia. Therefore, the developed hybrid plasmonic nanodiamonds were used as thermal agents for local photothermal therapy (PTT) against melanoma *in vivo*. This investigation demonstrates the efficacy of hybrid plasmonic nanodiamonds in photothermal eradication of cancer cells and temperature monitoring.

## 2 Results and discussion

**Figure 1:**
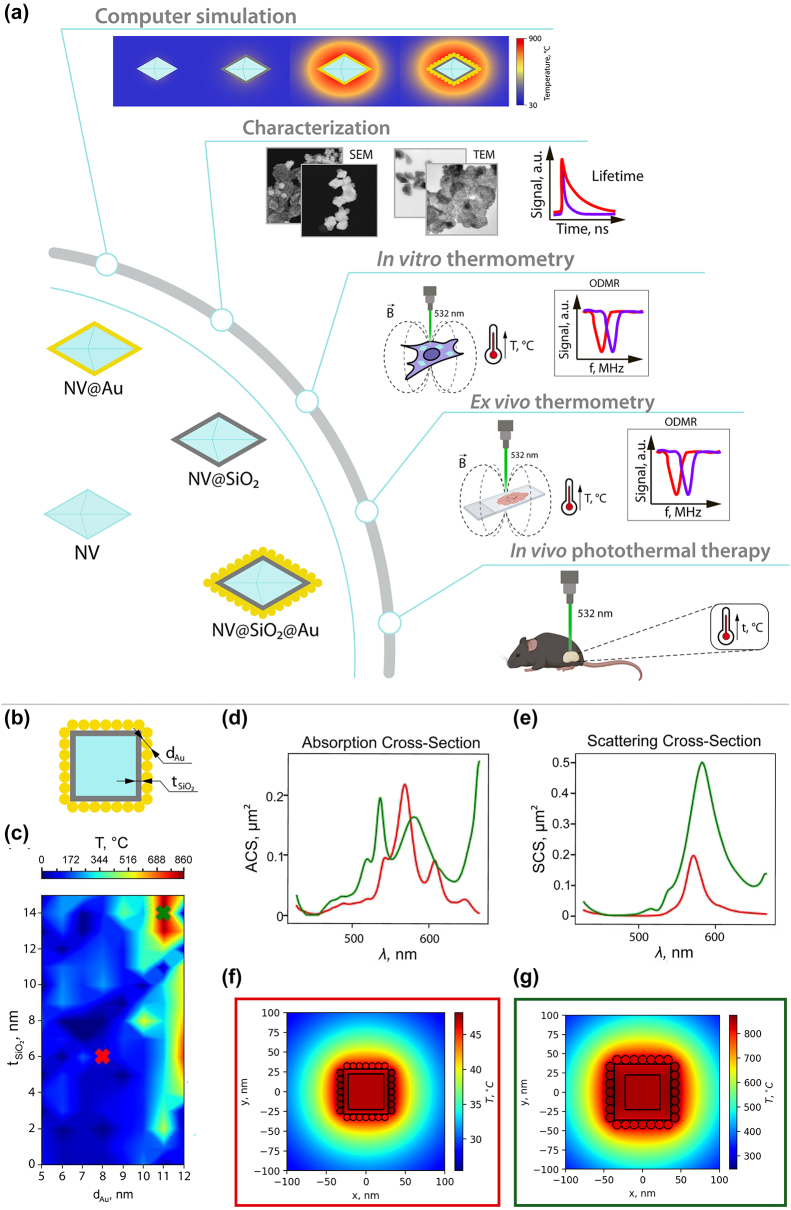
Road map and numerical simulation. (a) Road map of the study: (i) illustration of the obtained samples, (ii) numerical simulation of optical heating of plasmonic nanodiamonds, (iii) structural and optical characterization of the obtained samples, (iv) thermometry inside cells *in vitro*, (v) thermometry inside tissues *ex vivo*, (vi) photothermal therapy using plasmonic nanodiamonds *in vivo*. (b) Schematic illustration of the investigated nanostructure. (c) Heating map depending on the Au NPs’ diameter and thickness of SiO_2_ layer. Red cross corresponds to a non-resonant case with minimal heating and green cross to the resonant case. (d) Absorption cross-section for the resonant (green line) and non-resonant cases (red line). (e) Scattering cross-section for the resonant (green line) and non-resonant cases (red line). (f) Heat distribution for non-resonant nanostructure. (g) Heat distribution for resonant nanostructure.

The main milestones of this study are presented in Figure 1(a), which includes numerical simulation of heating abilities of hybrid plasmonic nanodiamonds, synthesis and characterization of the obtained nanodiamonds differently coated with Au NPs, their heating abilities on glass substrate, inside cells *in vitro*, and in tissues *ex vivo* with simultaneous thermometry, and use of the obtained plasmonic nanodiamonds as heating agents in local PTT of melanoma. The proposed novel and straightforward synthesis approaches allowed us to obtain hybrid plasmonic nanodiamonds either fully covered with an Au shell or densely coated with Au NPs. Rigorous investigation of the optical performances of the obtained plasmonic nanodiamonds proves their applicability as effective nanoheaters and as nanothermometers using a single excitation source with excellent sensitivity superior to other optical thermometry approaches [47]–[49].

### 2.1 Numerical simulation

First, we performed numerical simulations using CST Studio Suite to optimize the geometrical parameters of **NV@SiO**_**2**_ **@Au** nanostructures aiming to provide the highest absorption (highest temperature) at the pump wavelength (532 nm). In this structure, only Au absorbs light, which can be converted into heat. Generally, the real shape of nanodiamonds is polygonal and varies from sample to sample. In our simulation model, we used a “cube” as a shape for nanodiamonds. The used shape of nanodiamonds in the simulation model, however, does not play a significant role, since nanodiamonds do not contribute to the optical heating due to their thermal properties [50]. All the calculations were carried out in an aqueous medium (*n* = 1.33 in the considered wavelength range) [51], since in biomedical applications NPs are immersed in aqueous media. For this, we modelled a nanodiamond as a cube with a 45 nm side, covered with a SiO_2_ layer, with Au NPs on its surface (Figure 1(b)). We should note that pure nanodiamonds with such dimensions (<200 nm) have no resonance in the visible wavelength range. Then, we calculated the heating map of this nanostructure, varying two parameters: the thickness of the SiO_2_ layer, which is virtually the distance between the Au NPs and the NV surface, and the Au NPs’ diameter (Figure 1(c)). For excitation, a plane wave was used with a wavelength of 532 nm. The intensity of the plane wave was set to 1 MW/cm^2^. The SiO_2_ layer affects the resonance wavelength of the structure as shown in Figure 1(c). In terms of the heating process, neither silica nor nanodiamond has losses [50], [52], and only Au absorbs laser power and transforms it into heat. In the resonant case, the absorption peak wavelength of the structures coincides with the pump wavelength. Thus, in the heating map, we chose two points corresponding to the resonant (green cross) and non-resonant (red cross) cases to compare their absorption and scattering cross-sections (Figures 1(d) and 1(e)) and heat distribution (Figures 1(f) and 1(g)). In the non-resonant case (Figure 1(d), red curve), the absorption is red-shifted. In the numerically calculated scattering spectra, scattering peaks for resonant (Figure 1(e), green curve) and non-resonant (Figure 1(e), red curve) cases are located at almost the same wavelength. However, in the non-resonant case, the absorption peak overlaps with the scattering peak, and the scattering intensity decreases due to redistribution of energy between the absorption and scattering channels. We should note that while a large scattering cross-section is essential for visualization of such hybrid plasmonic nanomaterials, effective PTT at minimal laser power density requires an increased NP absorption cross-section with low scattering losses [53]. In addition, the difference in absorption and scattering cross-sections for resonant and non-resonant cases can be explained by different Au NPs’ diameters, since Au NPs’ shape, size and aggregation state strongly correlate with their plasmonic resonances [54], [55]. As a result, the samples are heated to a temperature 17 times higher (Figures 1(f) and 1(g)) in the resonant case (green square). Thus, by proper choice of the Au NPs’ diameter, the absorption maximum of the hybrid structure can be matched with the excitation of NV centers to provide effective heating with simultaneous temperature monitoring via nanodiamonds excitation.

Therefore, we have determined the optimal geometrical parameters of the nanostructure, matching the absorption peak with the excitation wavelength, to be *t*_SiO2_ = 14 nm and *d*_Au_ = 11 nm. As a result, the heating temperature increased 17 times at the same pump intensity, which makes these nanostructures promising for further application in PTT.

### 2.2 Synthesis of plasmonic nanodiamonds

**Figure 2:**
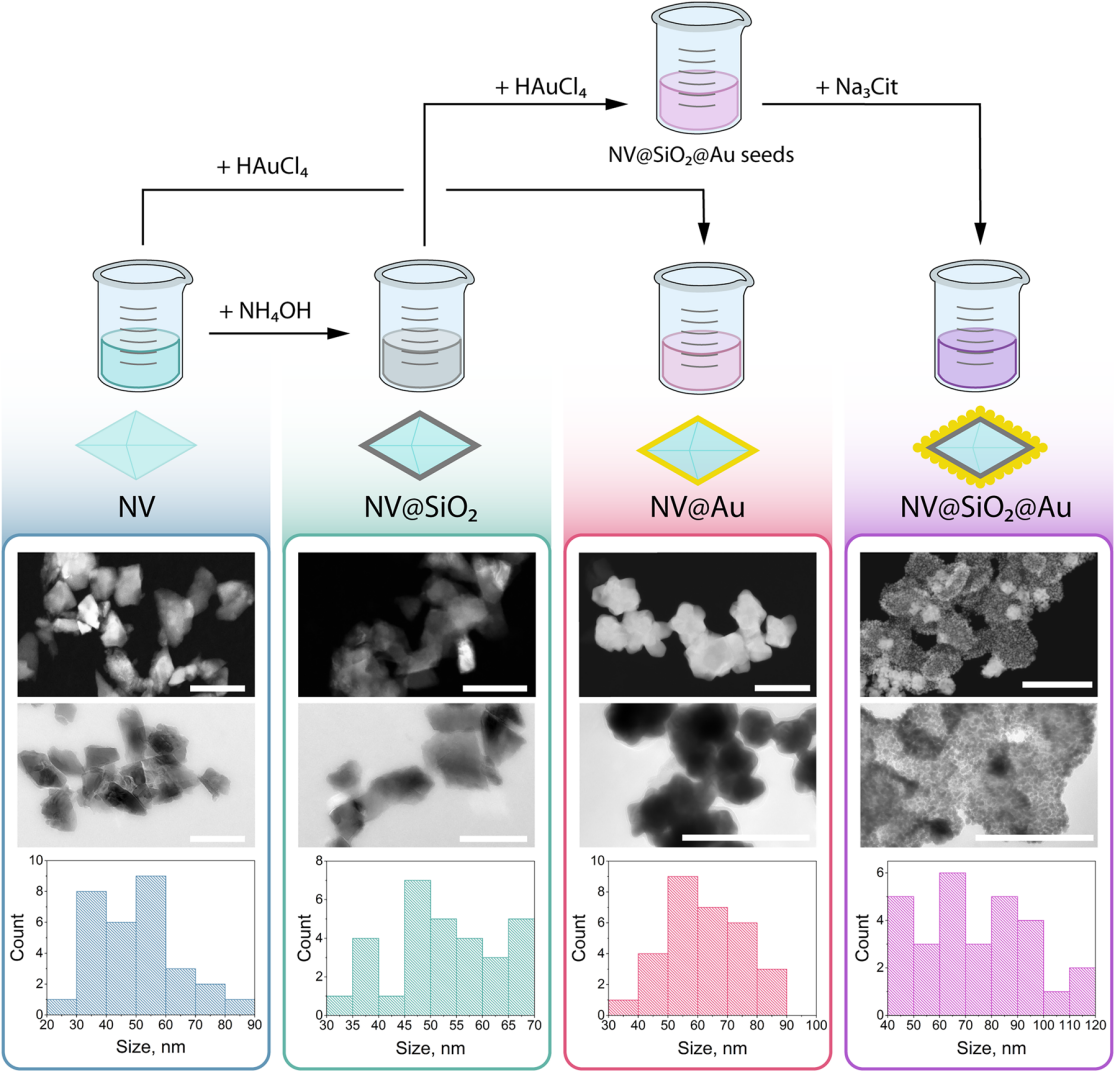
Synthesis and characterization of nanodiamond-based samples. Top: schematic illustration of synthesis of nanodiamond-based samples, from left to right: **NV**, **NV@SiO**_**2**_, **NV@Au**, and **NV@SiO**_**2**_ **@Au**. Bottom: representative transmission electron microscopy (TEM) images of nanodiamond-based samples with the corresponding size distributions derived from TEM obtained for (from left to right) **NV**, **NV@SiO**_**2**_, **NV@Au**, and **NV@SiO**_**2**_ **@Au**. Scale bar corresponds to 100 nm.

**Figure 3:**
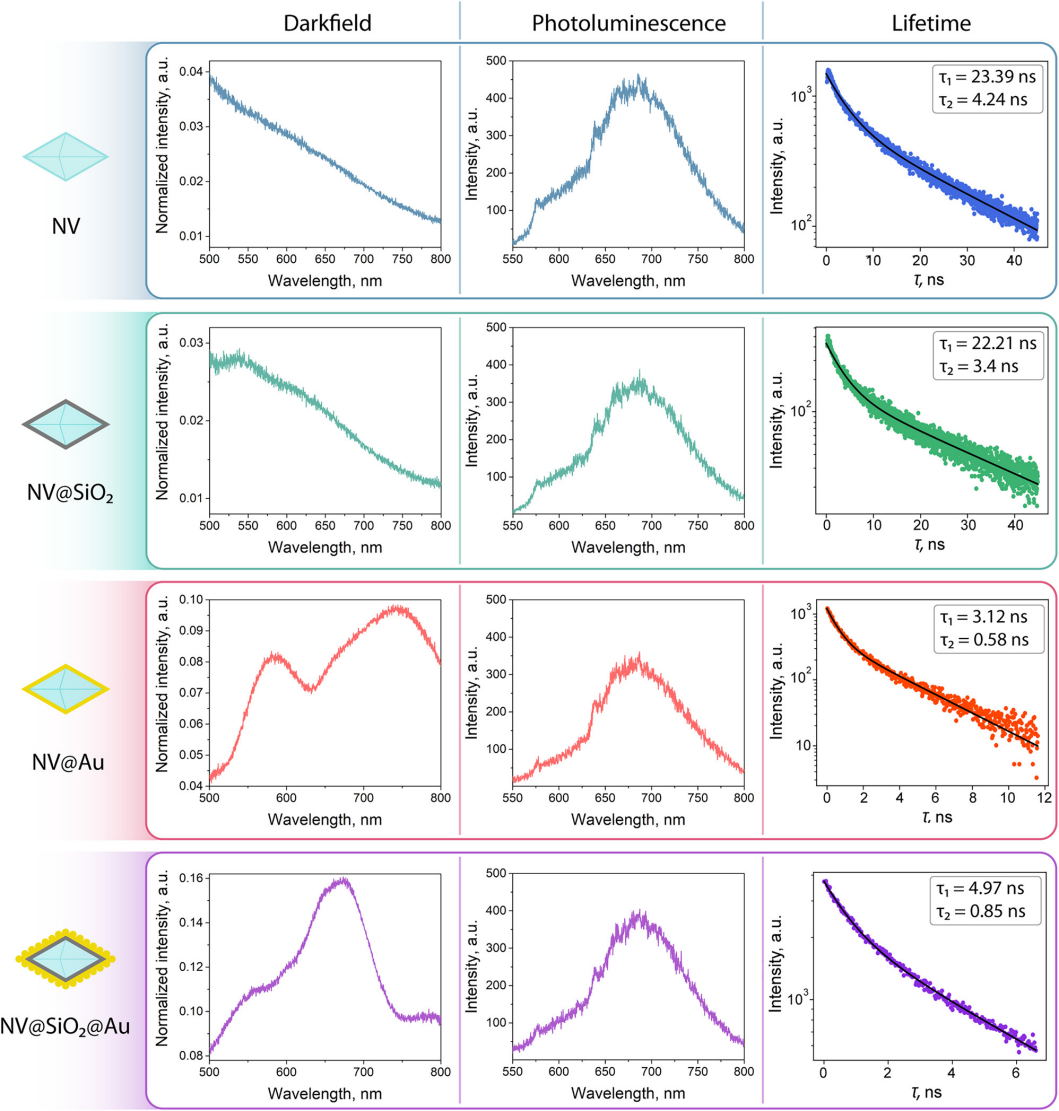
Optical characterization of **NV**, **NV@SiO**_**2**_, **NV@Au**, and **NV@SiO**_**2**_ **@Au**: *left column*: dark-field spectra, *middle column*: photoluminescence spectra, *right column:* lifetime spectra.

Commercially available nanodiamonds were coated with Au using different approaches to obtain either an Au shell or Au NPs attached on the surface of nanodiamonds. First, an Au shell was grown around fluorescent nanodiamonds by a one-step procedure using controlled reduction of the Au precursor [56]. In this approach, the surface of nanodiamonds serves as a nucleation site for the growth of Au shell, where the nanodiamonds can reduce Au(III) to metallic Au in the presence of hydroxylamine at pH 8.5 (**NV@Au**). Alternatively, for the coating with Au NPs, nanodiamonds were first covered with a silica shell with amino groups using (3-Aminopropyl)triethoxysilane (APTES) via sol−gel approach (NV@SiO_2_–NH_2_). Then, after transferring NV–SiO_2_–NH_2_ to HAuCl_4_, Au seeds were formed on the surface of silica-coated nanodiamonds via the reduction with NaBH_4_. Then, Au NPs were created on the surface of silica-coated nanodiamonds by further addition of the Na_3_Cit-to-HAuCl_4_ mixture with the following addition of hydroxylamine (**NV@SiO**_**2**_ **@Au**) [57]. Pristine nanodiamonds (**NV**) and nanodiamonds coated with silica shell (**NV@SiO**_**2**_) were used as control samples for the following experiments. In total, four different types of nanostructures were obtained (Figure 2). Notably, SiO_2_ shells shield nanodiamonds from external charges, and they also reduce the electric and magnetic noise on the surface of NV centers, which is vital for measuring ODMR spectra [28]. All the obtained NV-based samples were visualized with transmission electron microscopy (TEM), and the corresponding size distributions were derived from TEM images (Figure 3). According to the obtained data, the average sizes for **NV** were equal to 48.88 ± 9.83 nm; for **NV@SiO**_**2**_, to 52.48 ± 9.89 nm; for **NV@Au**, to 62.73 ± 12.71 nm; and for **NV@SiO**_**2**_ **@Au**, to 73.98 ± 20.64 nm. In the case of **NV@SiO**_**2**_ **@Au**, Au NPs, 8.97 ± 1.67 nm in size, almost completely covered the surface of NV, forming a layer due to preferential attachment of the Au seeds from the growth solution to the surface areas of the silica layer. Indeed, the electrostatic interactions between the Na_3_Cit-stabilized negatively charged Au seeds and the positively charged NH_2_-functionalized silica layer result in Au nanoshells consisting of densely packed Au NPs. EDX analysis revealed the presence of all the corresponding elements in the obtained samples (Figure S1). The hydrodynamic diameters of **NV**, **NV@SiO**_**2**_, **NV@Au**, **NV@SiO**_**2**_ **@Au** were measured in water after the synthesis (Figure S2).

### 2.3 Optical characterization of plasmonic nanodiamonds

Next, we investigated the fabricated nanostructures (**NV**, **NV@SiO_2_**, **NV@Au**, and **NV@SiO_2_@Au**) with optical methods to estimate their response properties and compare them with the numerical calculations (for **NV@SiO**_**2**_ **@Au)**. First, we measured the dark-field spectra of each structure. The optical scheme and experimental details are available in the Supporting Information (Figure S3). The dark-field scattering spectra of **NV@Au** and **NV@SiO**_**2**_ **@Au** are characterized by broadband plasmonic resonances. For instance, in the case of **NV@Au**, there are two peaks at 580 and 750 nm stemming from the shell morphology: non-uniform thickness of the Au shell (grey and black areas in Figure 2) and its angular shape can lead to the presence of two resonance peaks [58]–[60]. Furthermore, for **NV@SiO**_**2**_ **@Au,** a single peak at 650 nm is observed, attributed to the close proximity of Au NPs on the surface of nanodiamonds. The peak position for **NV@SiO**_**2**_ **@Au** is in agreement with the numerical modelling results, which demonstrated a pronounced peak near 600 nm for Au NPs attached onto the surface of nanodiamonds. As expected, scattering for **NV** and **NV@SiO**_**2**_ is relatively weak, and there are no pronounced peaks. In turn, there is noticeable scattering for hybrid plasmonic nanodiamonds (**NV@Au** and **NV@SiO**_**2**_ **@Au**)**,** featured due to the presence of Au clusters and their interparticle coupling [61]–[63].

Then, we estimated the fluorescence spectra for each tested sample (Figure S3). For their excitation, a 532 nm laser was used. The fluorescence spectra for all the nanostructures reveal a broad emission band between 550 and 750 nm, typical for pristine nanodiamonds. The zero-phonon line (ZPL), one of the narrowband peaks in the photoluminescence spectra originating from defects, which describes a spin state, is located at 638 nm for all the tested samples. This ZPL line is attributed to the negatively charged (NV–) state of the nitrogen vacancy center required for ODMR measurements [64], [65].

Finally, we estimated the fluorescence lifetime for each sample using time-correlated single photon counting (TCSPC) approach (Figure S4). For their excitation, a 532 nm laser was used. The obtained data was fitted biexponentially, relying on two characteristic times: a slower component *τ*_1_ and the faster one *τ*_2_, which provided a more precise fit. According to the obtained data, the fluorescence lifetimes of both components for **NV** and **NV@SiO**_**2**_ are almost equal, confirming that SiO_2_ coating does not significantly affect the optical properties of the nanodiamonds. However, fluorescence lifetime shortening of both components for **NV@Au** and **NV@SiO**_**2**_ **@Au** can be explained by an accelerated transition dynamics of NV centers. A possible reason is that in the created plasmonic nanocavity (Au shell), resonances arise due to the interparticle coupling between the plasmon and NV centers and spatial proximity of nanodiamond and Au [62], [66]. In case of **NV@SiO**_**2**_ **@Au**, coupling also occurs, since the thickness of the silica layer is low compared to Au; nonetheless, the Au-nanodiamond interaction is partially shielded by the silica layer, resulting in a slightly longer lifetime compared to **NV@Au** (4.97 ns vs. 3.12 nm for the longer component *τ*_1_, respectively).

Therefore, the research of the optical properties of the obtained nanodiamond-based structures has demonstrated that the Au shell layer and Au resonant particles lead to the appearance of the plasmonic resonance, as was demonstrated in numerical simulation. As a result, we assume that the observed plasmonic resonance shortened the NV centers’ emission lifetime.

### 2.4 Optical heating of plasmonic nanodiamonds *in vitro* and *ex vivo*

**Figure 4:**
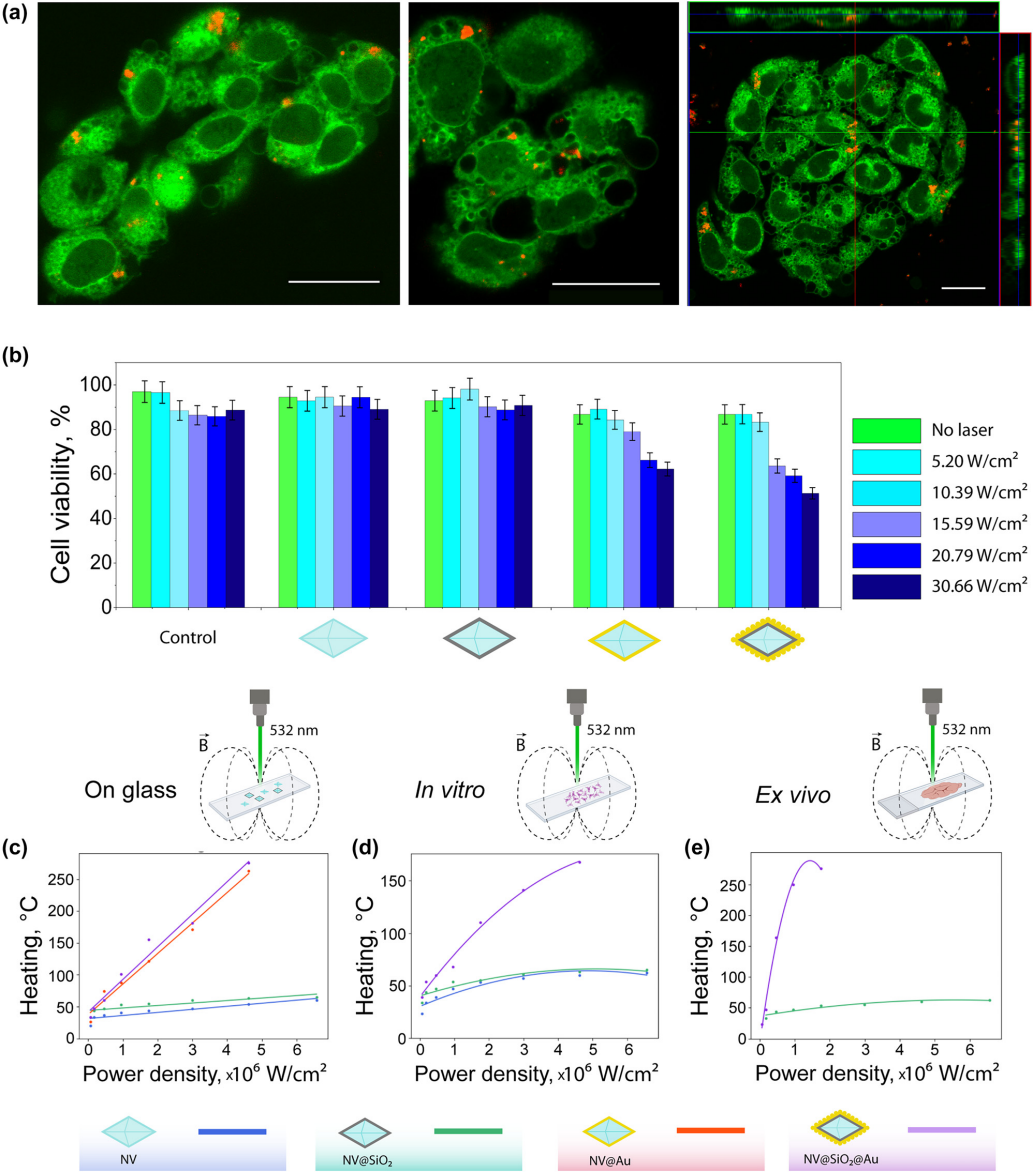
Cell studies and thermometry. (a) Representative confocal laser scanning microscopy (CLSM) images of B16-F10 cells (stained with rhodamine B, green fluorescent signal) with BSA-Cy5 labelled **NV@SiO**_**2**_ (red fluorescence signal). Right image: orthogonal views from different planes (*x*/*y*, *x*/*z*, and *y*/*z*) of a confocal microscope image used to visualize the particle uptake. Scale bar corresponds to 20 μm. (b) B16-F10 cell viability after incubation with 200 μg/mL of **NV**, **NV@SiO**_**2**_, **NV@Au**, and **NV@SiO**_**2**_ **@Au** irradiated with a 532 nm laser at different laser power densities (5.2 W/cm^2^, 10.39 W/cm^2^, 15.59 W/cm^2^, 20.79 W/cm^2^, and 30.66 W/cm^2^). (c) Experimentally measured temperature (heating) of dried **NV**, **NV@SiO**_**2**_, **NV@Au**, and **NV@SiO**_**2**_ **@Au** on glass depending on the applied power density. (d) Experimentally measured temperature (heating) of **NV**, **NV@SiO**_**2**_, and **NV@SiO**_**2**_ **@Au** associated with B16-F10 cells *in vitro* depending on the applied power density. (e) Experimentally measured temperature (heating) of **NV@SiO**_**2**_ and **NV@SiO**_**2**_ **@Au** inside tissue *ex vivo* depending on the applied power density.

Further, general interaction of the obtained plasmonic nanodiamonds with B16-F10 melanoma cells was investigated. Confocal laser scanning microscopy (CLSM) was used to check the ability of the **NV@Au** and **NV@SiO**_**2**_ **@Au** to be internalized by cells. For this, plasmonic nanodiamonds were additionally fluorescently stained with Cy5 dye and then incubated with B16-F10 cells pre-seeded in confocal dishes overnight. Afterwards, the cells were stained with Rhodamine B, and CLSM images were taken in 2D and 3D (Z-stack option) mode. Red fluorescence signal from the labeled nanodiamonds surrounded by green fluorescence signal from stained cells indicates intracellular localization of NPs (Figure 4(a)). After incubating the B16-F10 cells with all the nanodiamond-based samples (**NV**, **NV@SiO**_**2**_, **NV@Au**, and **NV@SiO**_**2**_ **@Au**) added at a concentration of 200 μg/mL for 24 h and irradiating them with a quasi-CW (80 MHz) 532 nm laser at different power densities (up to 30.66 W/cm^2^), the viability of the cells was assessed using Alamar blue test. Notably, the used 532 nm laser was applied for both optical heating and excitation of NV centers allowing significant simplification of experimental setup. In case of **NV** and **NV@SiO**_**2**_, no obvious toxicity was detected, since pristine nanodiamonds and nanodiamonds covered with silica are not heated without plasmonic structures. In contrast, in the case of plasmonic nanodiamonds (**NV@Au**, **NV@SiO**_**2**_ **@Au**), viability of B16-F10 cells decreased with increasing laser power density. For **NV@Au**, the percentage of viable cells was 62.2 %, and for **NV@SiO**_**2**_ **@Au**, 51.32 % at a higher applied laser power density (30.66 W/cm^2^).

The change in the cell viability can be attributed to the hyperthermia caused by optical heating of the plasmonic nanodiamonds. To monitor heat generation under laser irradiation, a home-built setup based on a confocal microscope supplemented with microwave and TCSPC modules was used (Figures S5 and 6). Representative measured and fitted ODMR spectra of NV at room temperature are shown in Figure S7. Heating abilities of all the obtained nanodiamond-based samples (**NV**, **NV@SiO**_**2**_, **NV@Au**, and **NV@SiO**_**2**_ **@Au**) were measured on glass using dried individual particles. For comparison, we measured the heat release from the obtained modified nanodiamonds associated with cells *in vitro* and in tissues *ex vivo*. For instance, in the case of *in vitro* thermometry in association with cells, **NV**, **NV@SiO**_**2**_, and **NV@SiO**_**2**_ **@Au** were incubated with B16-F10 cells overnight. Then, after thorough washing, cells with associated nanodiamond-based samples were concentrated via centrifugation, and generation of heat was consequently measured. Notably, the thermometry of the **NV@Au** sample in cells *in vitro* was not possible, since we assume that the Au coating induces charge migration from nanostructures to cells and spin depolarization by charge-state conversion [67]. For *ex vivo* thermometry, **NV@SiO**_**2**_ and **NV@SiO**_**2**_ **@Au** were intratumorally injected into B16-F10 melanoma-bearing C57BL/6 mice. After 24 h, the tumors were extracted and sliced, and ODMR spectra from the tissue slices containing **NV@SiO**_**2**_ and **NV@SiO**_**2**_ **@Au** were measured under irradiation using the same laser at different power densities. In case of *ex vivo* thermometry with nanodiamonds, **NV** and **NV@Au** were not considered due to the presence of external charges inside tissues, whereas the silica layer in **NV@SiO**_**2**_ **@Au** preserves nanodiamonds from solvents and polarity of the surrounding environment [68]. Importantly, in case of thermometry, the used laser power densities were up to 6.5 × 10^6^ W/cm^2^, i.e. higher than in cell viability measurements due to a smaller laser spot. According to the obtained results, the highest heat release was obtained from **NV@SiO**_**2**_ **@Au** (276 ± 1.76 °C) followed by **NV@Au** (263 ± 1.7 °C), which correlates with the numerical calculation results and the presence of a plasmonic coating. As expected, pristine nanodiamonds and silica-coated nanodiamonds showed no heating at the applied laser power densities due to absence of material losses in the visible wavelength range [52]; as a result, the silica layer did not impact the temperature evaluation. Interestingly, the level of heat generation in tissue slides was similar to that on glass (276 ± 1.76 °C); however, in case of *in vitro* cell thermomery, plasmonic nanodiamonds heated up to 168 ± 1.76 °C. This can be attributed to the presence of aqueous medium and the corresponding dissipation of the optical signal [5], [69], [70]. Examples of fitted ODMR spectra corresponding to different temperatures are shown in Figure S8. Slightly decreased thermometry sensitivity can be attributed to the presence of Au NPs or Au shell on the surface of NV centers that decrease the thermometry performance, since the coverage of NV centers with Au structures leads to the non-optimal resonance of the microwave antenna used for measurements, which was designed to match with the ODMR peak at room temperature. It should be highlighted that for the case of the glass substrate the fitting curve was linear. However, in case of *ex vivo* measurements the process of heating was not linear, and polynomial approximation was more suitable due to non-uniform heat transfer. The optical heating process may lead to coagulation, ablation or welding of cells and tissues depending on operating conditions such as laser wavelength, beam profile and spatial heat distribution, exposure duration [71], [72]. It should be noted that the heat generation occurs at nanoscale, not affecting the entire cell or tissue volume.

### 2.5 *In vivo* biodistribution of plasmonic nanodiamonds

The obtained plasmonic nanodiamonds were designed for localized treatment, similar to other nanoplatforms undergoing evaluation in preclinical and clinical studies [73]–[76]. To ensure their suitability for PTT, we investigated their retention within tumors during 24 h. Additionally, we included a group of mice receiving a diuretic (furosemide) to mimic a clinical scenario of patients taking such medications. Furosemide, commonly used for edema and hypertension unresponsive to other treatments [77], was evaluated for its potential impact on the plasmonic nanodiamonds’ retention in the tumor tissue. To corroborate this data, an additional control group of dehydrated mice (the animals were deprived of water for a few hours) was included. Dehydration is a prevalent condition among the elderly, and it can significantly impact the vascular system [78]. This is particularly crucial in cancer research, as many elderly patients may experience both conditions.

**Figure 5:**
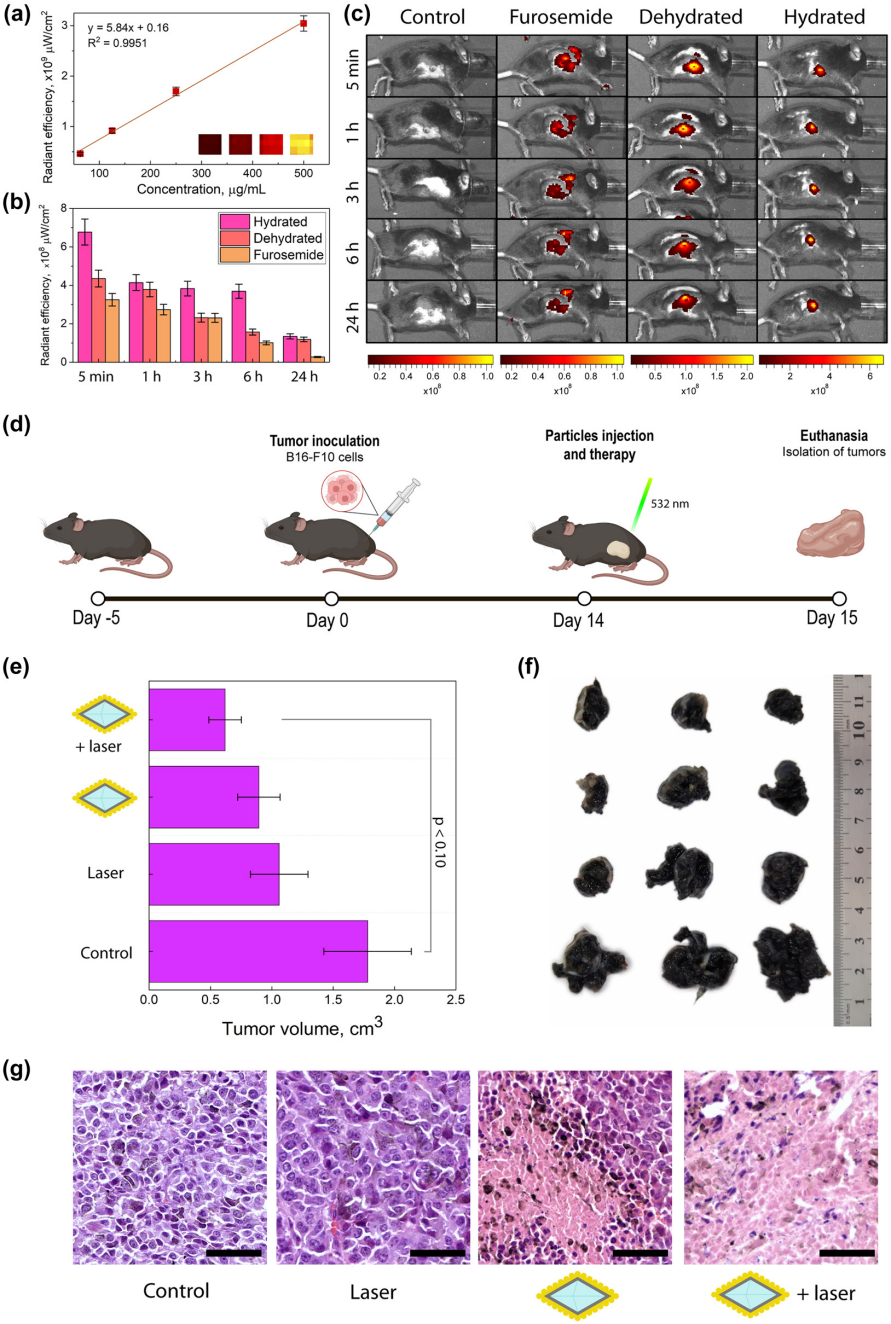
*In vivo* studies. (a) Fluorescence signal of Cy5-labeled **NV@SiO**_**2**_ phantoms at different concentrations. (b) Fluorescence signals obtained from regions of interest derived from *in vivo* images of hydrated mice, dehydrated ones, and those treated with furosemide with injected **NV@SiO**_**2**_. (c) *In vivo* fluorescence images of mice that were hydrated, dehydrated, treated with furosemide, or unaffected (control group), 5 min, 1 h, 3 h, 6 h, and 24 h after intratumoral injection of **NV@SiO**_**2**_. (d) Scheme of therapy and tumor growth estimation. (e) Measured tumor volumes after PTT (significant difference threshold was defined as *p* ≤ 0.10 which presents a statistical tendency of an observed therapeutic effect). (f) Digital photos of tumors extracted after the therapy. (g) H&E-stained histological images of tumors after treatment with **NV@SiO**_**2**_ **@Au** with laser, or **NV@SiO**_**2**_ **@Au**, or control with laser, or control with 0.9 % NaCl. Scale bars correspond to 50 µm. All the error bars show the standard deviation determined from three independent assays.

To validate the *in vivo* retention of plasmonic nanodiamonds within the tumor, we used the B16-F10 tumor-bearing C57BL/6 mouse model, which was established by subcutaneous injection of B16-F10 cells. Due to the similar sizes of our nanodiamond-based samples, we used only the Cy5-labelled **NV@SiO**_**2**_. At first, these NPs were used for the formation of aqueous phantoms to evaluate the contrast properties. For this, phantoms of different concentrations up to 500 μg/mL were imaged using a fluorescence imaging system, IVIS Spectrum CT (Perkin Elmer), at 675 nm and 720 nm excitation/emission wavelengths. Cy5-labelled **NV@SiO**_**2**_ nanodiamonds provided fluorescence signals that increased with increasing concentration (Figure 5(a)). Before the imaging, mice were divided into three groups (*n* = 5): (i) hydrated mice, (ii) dehydrated mice, and (iii) hydrated mice with furosemide introduction (single dose of 200 mg/kg, *per os*, 1 h before the injection of NPs). Afterwards, **NV@SiO**_**2**_ were intratumorally administered into mice (100 μL, 500 μg/mL), and the mice were imaged with the IVIS imaging system (Figure 5(a)). Based on the obtained data, fluorescence signal from the nanodiamonds was found in tumors and decreased with time after the **NV@SiO**_**2**_ injection. This can be explained by the degradation of nanodiamonds, which can lower the fluorescence intensity within the tumor. Overall, the hydrated samples showed a higher fluorescence signal intensity compared to that originating from the mice that were dehydrated or treated with furosemide (fluorescence intensity was 20 %, 18 % and 10 % 24 h after the injection, respectively) (Figures 5(b) and 5(c)).

Further, we proceeded with the therapy studies using **NV@SiO**_**2**_ **@Au** intratumorally introduced into B16-F10 melanoma-bearing C57BL/6 mice.

### 2.6 Photothermal therapy using plasmonic nanodiamonds

Photothermal therapy was performed on four groups of animals: (i) **NV@SiO**_**2**_ **@Au** with laser, (ii) **NV@SiO**_**2**_ **@Au**, (iii) control with laser, and (iv) control with 0.9 % NaCl. The amount of injected **NV@SiO**_**2**_ **@Au** was 100 μg, 200 μg/mL. After a tumor was developed in a mouse, **NV@SiO**_**2**_ **@Au** were injected directly into the tumor, since the targeting abilities of the obtained plasmonic nanodiamonds are absent per se. After the injection, all the control groups were irradiated with a 532 nm laser for 5 min (30.66 W/cm^2^) (Figures 5d and S11). The next day, mice were sacrificed, and the geometrical sizes of the tumors were measured (Figures 5(e) and 5(f)). As expected, injecting the plasmonic nanodiamonds alone or irradiating tumors with laser without plasmonic nanodiamonds (controls) resulted in a slight inhibition of tumor growth, by 40.45 % and 49.94 %, respectively. Notably, inhibition of tumor growth was more pronounced after the injection of the plasmonic nanodiamonds and consequent laser irradiation, by 65.22 %. This can be attributed to the release of heat from **NV@SiO**_**2**_ **@Au** at nanoscale, up to 168 °C *in vitro* and 276 °C *ex vivo* (Figure 4(d)), which leads to cell hyperthermia. Generally, elevated temperatures reached during PTT reduce tumor volumes, which correlate with the obtained data. We should also highlight that the PTT in this study, being a proof-of-concept, was performed at non-optimal conditions: above all, the used light source (Coherent Obis, 532 nm, 30.66 W/cm^2^) is not well-suited for biological tissues due to optical signal dissipation from a heterogeneous environment [5], [69], [70]. Therefore, we had to increase the laser power density, which slightly affected the tumor cells in the group of mice not injected with **NV@SiO**_**2**_ **@Au**. This was further confirmed by histological analysis of tumors from all the groups that experienced the laser irradiation.

Tumor cells in the control group (0.9 % NaCl) were polymorphic, polygonal and spindle-shaped. Numerous accumulations of melanin granules were observed, and cells in the mitotic stage were also found. Vascularization was also present in the studied tumors. Invasive tumor growth and infiltration of the surrounding healthy tissues, i.e. the dermis and striated skeletal muscle, were noted. For the case of intratumoral administration of **NV@SiO**_**2**_ **@Au**, the histological picture was similar to that in the control group, which indicates the biocompatibility of the used plasmonic nanodiamonds. For tumor samples exposed to laser irradiation without **NV@SiO**_**2**_ **@Au** injection, small or fine confluent areas of necrosis are observed. The volume of necrotic areas ranges from 30 % to 40 % of the total tumor volume, which is in agreement with the fact that we had to increase the laser power density affecting cells. There is also a decrease in the number of cells in the mitosis stage. In the tumors from the mouse group treated with laser, the accumulation of melanin in tumor cells decreased, and its release and accumulation in the intercellular space increased due to violation of the cellular structure. The described effects are enhanced for the injection of plasmonic nanodiamonds combined with laser irradiation. Large necrotic areas were observed, ranging from 50 % to 70 % of the total tumor volume.

Our work describes a straight-forward and fast synthesis route for the synthesis of plasmonic nanodiamonds completely coated with an Au layer. On the other hand, careful optimization of synthesis parameters yielded an even distribution of Au NPs on the surface of nanodiamonds. Our detailed optical characterization of the nanodiamond-based samples revealed that Au coating preserves the photoluminescence properties of pristine nanodiamonds, but leads to their lifetime shortening due to accelerated transition dynamics. We have comprehensively studied the heating abilities of nanodiamonds with different Au coatings in various environments: on glass, associated with cells *in vitro*, and in tissues *ex vivo*. As mentioned, we applied a 532 nm laser for the excitation of NV centers, which does not fall within the transparency window for biological tissues. For this reason, the proposed thermometry approach is suitable for rather transparent animals such as zebrafishes, worms etc. [79]–[81], or for the case of *ex vivo* samples such as demonstrated in this study. As a proof of concept, the obtained plasmonic nanodiamonds were applied as heating agents for PTT of melanoma, proving the multifunctionality of the obtained platform. These findings underline the versatility of the proposed plasmonic nanodiamonds and offer exciting prospects for their future translation into clinical practice.

## 3 Experimental section

### 3.1 Numerical simulation

Numerical calculations of ACS, SCS, and heating were performed using CST Studio Suite. The detailed simulation procedure is presented in Supporting Information.

### 3.2 Synthesis of NV@SiO_2_, NV@Au, NV@SiO_2_@Au

All fabrication procedures were carried out using commercially available nanodiamonds. The NV centers were modified with a silica shell (**NV@SiO**_**2**_) using the Stöber method. **NV@Au** was obtained by a one-step procedure using reducing Au(III) to metallic Au in the presence of hydroxylamine at pH 8.5. **NV@SiO**_**2**_ **@Au** was synthesized by forming Au NPs grown from seeds on silica-coated nanodiamonds via the reduction with NaBH_4_. The details are provided in Supporting Information.

### 3.3 Structural characterization

The obtained nanostructures were characterized with scanning electron microscopy (SEM) and transmission electron microscopy (TEM). The characterization procedures for all the synthesized materials are presented in Supporting Information.

### 3.4 Optical characterization

The dark-field, fluorescence and lifetime measurements were performed using home-built optical setups. The schemes of the experimental setups are shown in Supporting Information.

### 3.5 Thermometry measurements on glass, in vitro and ex vivo

Temperature measurements in agarose and inside living cells were performed using optically-detected magnetic resonance under continuous wave microwave excitation. The scheme of the experimental setup is shown in Supporting Information.

### 3.6 Cell culture

Murine melanoma cell line (B16-F10 cells) was used for *in vitro* thermometry studies *and* toxicity studies. B16-F10 cells were obtained from the American Type Culture Collection and cultured with AlphaMEM supplemented with 10 % of vol. fetal bovine serum and 2 mM of UltraGlutamine I at 37 °C.

### 3.7 Toxicity study

The toxicity of the nanomaterials and the impact of laser irradiation were estimated using the AlamarBlue assay. The procedure is described in Supporting Information.

### 3.8 Animal studies

For *in vivo* studies, healthy C57BL/6 mice (males, 8-week-old, 18–22 g) were purchased from the St. Petersburg center of laboratory animals “Rappolovo” of the National Research Center “Kurchatov Institute” and were used for *ex vivo* and *in vivo* experiments, namely, *ex vivo* thermometry, biodistribution and therapy studies. Animals were maintained in accordance with the Guidelines for Accommodation and Care of Animals (European Convention for the Protection of Vertebrate Animals Used for Experimental and Other Scientific Purposes) and internal guidelines, and the experimental procedures were approved by the local authorities. The details are provided in Supporting Information.

### 3.9 Biodistribution experiments

For biodistribution investigation, mice with tumors were divided into 3 groups: (i) hydratated mice, (ii) dehydrated mice, and (iii) hydrated mice with furosemide introduction. Then, NV@SiO_2_ labelled with Cy5 were intratumorally injected (100 μL, 500 μg/mL). Animals were anaesthetized with isoflurane and imaged using IVIS Spectrum CT (Perkin Elmer) at 640/680 nm excitation/emission wavelengths after 1–24 h after the of NV@SiO_2_ injection. The details are provided in Supporting Information.

### 3.10 PTT studies

To perform PTT, mice were divided into 4 groups: (i) with intertumoral injection of NV@SiO_2_@Au (100 μg, 200 μg/mL) and irradiation with laser, (ii) with intertumoral injection of **NV@SiO**_**2**_ **@Au** without laser irradiation, (iii) only laser irradiation, and (iv) without laser irradiation. For PTT, each mouse was located under a perpendicular directed laser beam of a continuous wave laser (Coherent Obis, 532 nm, 30.66 W/cm^2^). Mice were irradiated for 5 min and left for 24 h. The details are provided in Supporting Information.

## Supplementary Material

Supplementary Material Details
