# Supplementary material for: Hybrid plasmonic nanodiamonds for thermometry and local photothermal therapy of melanoma: a comparative study
Source: Nanophotonics. 2024 Aug 28;13(22):4111–25. doi: 10.1515/nanoph-2024-0285 (PMC11501064; doi:10.1515/nanoph-2024-0285)
Supplement: Supplementary file 1 — Supplementary Material Details [file j_nanoph-2024-0285_suppl_001.docx]

**Supporting information**

**Hybrid plasmonic nanodiamonds for thermometry and local photothermal therapy of melanoma: a comparative study**

Elena N. Gerasimova^1^, Landysh I. Fatkhutdinova^1^, Ivan I. Vazhenin^1^, Egor I. Uvarov^1^, Elizaveta Vysotina^1^, Lidia Mikhailova^1^, Polina A. Lazareva^2^, Dmitry Kostyushev^3,4,5^, Maxim Abakumov^2,6^, Alessandro Parodi^7^, Vitaly V. Yaroshenko^1^*, Dmitry A. Zuev^1^*, Mikhail V. Zyuzin^1^*

^1^School of Physics and Engineering, ITMO University, Lomonosova 9, 191002 St. Petersburg, Russia

^2^Department of Medical Nanobiotechnology, N.I. Pirogov Russian National Research Medical University, Ostrovityanova 1 bldg. 6, 117997 Moscow, Russia

^3^Martsinovsky Institute of Medical Parasitology, Tropical and Vector-Borne Diseases, Sechenov University, Moscow, Russia

^4^Division of Biotechnology, Scientific Center for Genetics and Life Sciences, Sirius University of Science and Technology, Sochi, Russia

^5^Faculty of Bioengineering and Bioinformatics, Lomonosov Moscow State University, Moscow, Russia

^6^Laboratory of Biomedical Nanomaterials, National University of Science and Technology (MISIS), Leninskiy prospekt 4, 119049 Moscow, Russia

^7^Sirius University of Science and technology, Olympic Ave, 1, 354340 Nizhneimeretinskaya Bukhta, Krasnodarskiy kray

*corresponding authors: v.yaroshenko@metalab.ifmo.ru, d.zuev@metalab.ifmo.ru, mikhail.zyuzin@metalab.ifmo.ru

Numerical simulation

- Numerical simulation of heating
- Numerical simulation of absorption and scattering

Materials

- For nanodiamonds’ coating
- For cell cultures
- For animal studies

Synthesis of nanomaterials

- Synthesis of **NV@SiO_2_**
- Synthesis of **NV@Au**
- Synthesis of **NV@SiO_2_@Au**
- Synthesis of **NV@SiO_2_-Cy5**

Structural characterization of nanomaterials

- Transmission electron microscopy (TEM)
- Energy-dispersive X-ray spectroscopy
- Dynamic light scattering (DLS)

Optical properties of nanomaterials

- Photoluminescence and dark-field measurements
- Lifetime measurements
- Optically-detected magnetic resonance measurements
- The working principle of optically detected magnetic resonance (ODMR)
- Temperature determination and statistical analysis

Measurements of nanomaterials heating on glass

*In vitro* experiments

- Cells
- Toxicity studies
- Confocal laser scanning microscopy (CLSM)
- Temperature measurements inside cells

*Ex vivo* and *in vivo* studies

- Animals
- Tumor establishment
- Cryogenic histological sample preparation
- Temperature measurements ex vivo
- Biodistribution experiments
- Photothermal therapy (PTT)
- Histological analysis

**Numerical simulations**

- **Numerical simulations of heating**

First, we calculated the power absorbed by the considered structures in water, using the commercially available software CST Studio Suite. We considered a structure as a core-shell nanoparticle whose core consisted of a cube-shaped nanodiamond and the shell was made of SiO_2_ and Au NP layers with different thicknesses (0-15 nm) and diameters of NPs (5-12 nm). Nanostructures were irradiated by a linearly polarized plane wave with an intensity of about 1 MW/cm^2^. Next, the absorbed power was converted into heat, using the unidirectional mode of the EM-thermal coupling module in the steady-state regime. The optical properties of nanodiamonds were taken from the Ref.^1^, of gold from the Ref.^2^, of SiO_2_ from the Ref.^3^, and of water from the Ref.^4^. All the calculations were carried out in water, which was considered a lossless medium.^4^

In the thermal solver, we used material properties shown in the following table:

**Table S1**: Material properties for modeling

| **Material** | **thermal conductivity 𝜿, W K^-1^m^-1^** | **Specific heat capacity Cp, J kg^-1^K^-1^** | **References** |
| --- | --- | --- | --- |
| Nanodiamonds | 2000 | 520 | ^5^ |
| Au | 126 | 318 | ^6^ |
| SiO_2_ | 1.5 | 680 | ^7^ |
| Water | 0.6 | 4186 | ^6,8^ |

- **Numerical simulations of absorption and scattering**

Using numerical modeling of heating, we estimated the absorption and scattering cross-sections (ACS and SCS) of the structures for resonant and nonresonant cases using the commercially available CST Studio Suite. All the calculations were performed in aqueous medium (n=1.33 in the wavelength range of 400-700 nm).

**Materials**

- **For nanodiamonds’ coating**

NV centers (Carboxylated 60 nm Fluorescent Nanodiamond in DI water, <1 ppm NV, 1mg/ml) were purchased from Adamas Nanotechnologies. For SiO_2_ coating, tetraethyl orthosilicate (TEOS, MW = 208.33, 99.9%) and ethanol (C_2_H_5_OH, 95%) were obtained from Sigma-Aldrich. Ammonia solution (NH_3_×H_2_O, 28–30%) was purchased from Merck. For gold coating, gold (III) chloride trihydrate (HAuCl_4_·3H_2_O, ≥99.9%, Sigma-Aldrich), ascorbic acid (AA, ≥99.0%, Sigma-Aldrich), sodium borohydride (NaBH_4_, 98%, Sigma-Aldrich), (3-Aminopropyl)triethoxysilane (APTES, 99%, Merck), hydroxylamine hydrochloride ([NH3OH]Cl, 99%, Lenreactiv), and cetyltrimethylammonium bromide (CTAB, ≥99%, Sigma-Aldrich) were used as received. To modify NPs with dye, sulfo-cyanine 5 NHS ester (Cy5, M_W_ = 777.95) was obtained from Lumiprobe (Germany). Bovine serum albumin (BSA, 66 kDa) was bought in CSL Behring (Switzerland).

- **For cell cultures**

Alpha Minimum Essential Medium (Alpha-MEM) was purchased from Biolot, Russia. Phosphate-buffered saline (PBS), and UltraGlutamine I were purchased from Lonza, Switzerland. Antibiotic/Antimycotic Solution (100x) containing penicillin, streptomycin, and amphotericin B was purchased from Capricorn scientific, Germany. Fetal bovine serum (FBS) was obtained from HyClone, USA. TrypsinEDTA solution was purchased from Capricorn Scientific, Germany. Rhodamine B (RhB, ≥95%) and calcein acetoxymethyl (Calcein AM) were purchased from SigmaAldrich. AlamarBlue cell viability reagent was purchased from Invitrogen, USA.

- **For animal studies**

Zoletil (tiletamine hydrochloride 25 mg, zolazepam hydrochloride 25 mg) was purchased from Vibrac animal health, (India). Xyla (xylazine 20 mg) was purchased from Interchemie (The Netherlands). Saline solution (sodium chloride 0.9%) was purchased from Gematek (Russia).

**Synthesis of nanomaterials**

- **Synthesis of NV@SiO_2_**

For coating nanodiamonds with silicon dioxide, a modified Stöber method was used.^9,10^ For this, 500 μL (1 mg/mL) of nanodiamonds solution was diluted in 1.875 mL of ethanol. The resulting solution was placed in an ultrasonic bath and all the following steps were conducted under sonication. Then, 37.5 μL of ammonia solution was added to the nanodiamonds. Afterwards, 7.5 μL of TEOS were dropwise added to the reaction. The obtained mixture was sonicated for 1 h at 450 mW. The resulting silica-coated nanodiamonds were then washed 3 times with water (5 min, 11000 rpm). Afterwards, **NV@SiO_2_** were dispersed in 1 mL of water.

- **Synthesis of NV@Au**

For the synthesis of **NV@Au**, the modified previously reported protocol was used.^11^ In particular, 50 μL (1 mg/mL) of nanodiamonds were diluted in 4.95 mL of water. Then, 400 μL of 10 mM HAuCl_4_ was added, and the obtained suspension was stirred for 2 min at room temperature. Afterwards, 250 μL of 500 mM of reducing agent [NH_3_OH]Cl was mixed with nanodiamonds. To start the gold reduction, pH of the suspension was increased up to 8.5 by addition of NaOH at a pH of 12. When the pH of the suspension reached 8.5, it changed its color from white to pale red. Afterwards, pH value was increased once again up to 10. Then, 10 mM of HAuCl_4_ was added dropwise under stirring at room temperature until the suspension turned from pale red to grey-blue. The resulting suspension was then washed 3 times with water (10 min, 12000 rpm). After that, **NV@Au** were dispersed in 1 mL of water.

- **Synthesis of NV@SiO_2_@Au**

To obtain **NV@SiO_2_@Au,** the modified previously reported synthesis protocol was applied.^12^ First, NV@SiO_2_ with NH_2_ functional groups were first synthesised. For this, 500 μL of nanodiamonds solution (1 mg/mL) was diluted in 1.875 mL of ethanol. The resulting solution was placed in an ultrasonic bath, and all the following steps were conducted under sonication. Then, 37.5 μL of ammonia solution was added to the nanodiamonds. Afterwards, 7.5 μL of TEOS and 10 μL of (3-Aminopropyl)triethoxysilane (APTES) were slowly added to the nanodiamonds. The obtained reaction was sonicated for 1 h at 450 mW. The resulting solution was then washed 3 times with water (5 min, 11000 rpm). After that, NV@SiO_2_-NH_2_ were dispersed in 2 mL of water.

At the next step, NV@SiO_2_-NH_2_ were covered with Au seeds. For this, 2 mL of NV@SiO_2_−NH_2_ particles were mixed with 20 mL of 0.25 mM solution of HAuCl_4_. Then, 0.1 mL of 0.17 M solution of Na_3_Cit was added under stirring at 4 °C. Immediately after that (within 5 s), 1 mL of freshly prepared 0.01 M solution of NaBH_4_ was injected into the suspension, which was accompanied by an instant color change from pale yellow to reddish brown. After 6 min, the resulting nanoparticles were washed 1 time (10 min, 4000 rpm) and then redispersed in 3.6 mL of water.

Next, 0.4 mL of the seed-coated nanodiamonds were added to the 3.6 mL of mixture of 0.3 mM solution of HAuCl_4_ and 10.8 mM solution of Na_3_Cit. Afterwards, 39 μL of 40 mM solution of [NH_3_OH]Cl was added to the suspension. The resulting solution was then washed 3 times with water (5 min, 11000 rpm).

- **Synthesis of NV@SiO_2_ labeled with Cy5**

First, Cy5 dye was preliminary conjugated with bovine serum albumin (BSA) as reported elsewhere.^13^ Briefly, 77 mg of BSA was mixed with PBS. Further, 5 mg of Cy5 was diluted to 2.2 mL of DMSO and added to the resulting BSA solution. The prepared mixture was stirred at 4 °C for 24 h. Finally, to remove the unreacted Cy5 dye, a dialysis procedure was carried out for 72 h.

Then, to coat the nanodiamonds with SiO_2_ and Cy5, a modified Stöber method was used. For this, 500 μL of nanodiamonds solution was diluted in 1.875 mL of ethanol. Then, 37.5 μL of ammonia solution and 250 μL of Cy5-BSA was added to the nanodiamonds mixture under sonication. Afterwards, 7.5 μL of TEOS were slowly added. Obtained solution was sonicated for 1 h at 450 mW. The resulting mixture was then washed 3 times with water (5 min, 11000 rpm) and dispersed in 1 mL of water.

**Structural characterization of nanomaterials**

- **Transmission electron microscopy (TEM)**

Before the measurements, 1 µL of the nanodiamonds, either coated or pristine, were dropped on copper grids and dried. The geometry of the obtained nanodiamond-based samples was characterized by a transmission electron microscope, LIBRA 200 FE HR, Carl Zeiss, with an accelerating voltage of 200 kV.

- **Energy-dispersive X-ray spectroscopy (EDX)**

Elemental maps were imaged with TEM Zeiss Libra 200FE equipped with a JEOL Centurio energy-dispersive 1sr X-ray spectrometer (EDXS). The EDX spectra were processed using JEOL Analysis Station software. The obtained elemental maps are shown in **Figure S1**.


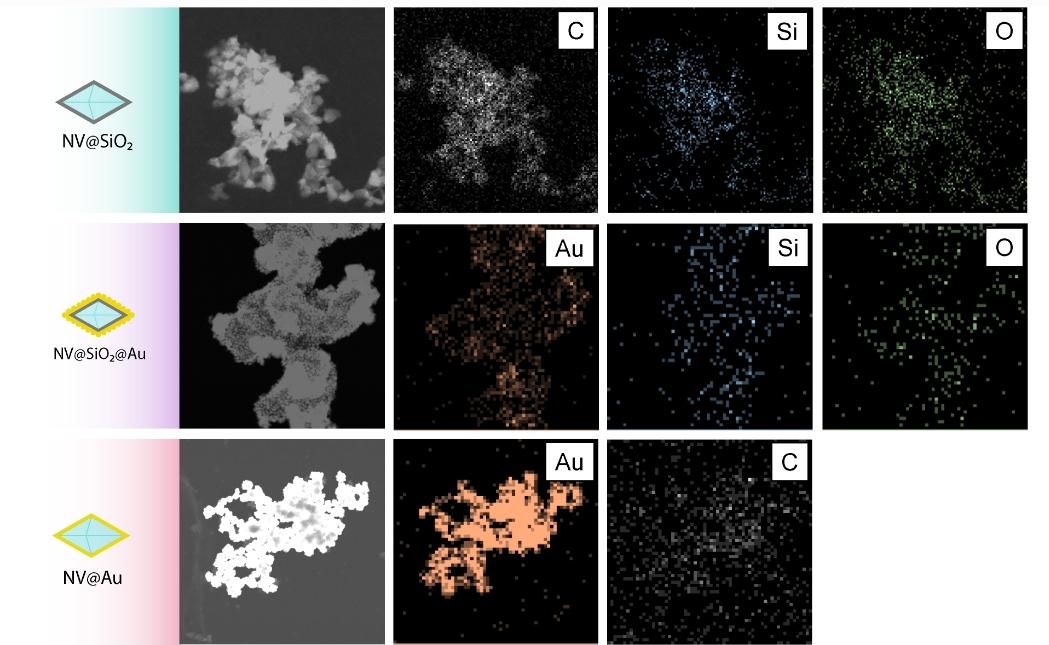


**Figure S1.** EDX elemental mapping of **NV@SiO_2_**, **NV@SiO2@Au,** **NV@Au**.

- **Dynamic light scattering (DLS)**

Dynamic light scattering was used to verify the hydrodynamic diameters of the synthesized nanomaterials. Their hydrodynamic diameters (Dh, nm) were measured using a Photocor Complex particle size analyzer (Photocor, Russia) equipped with a semiconductor laser operating at 638 nm.


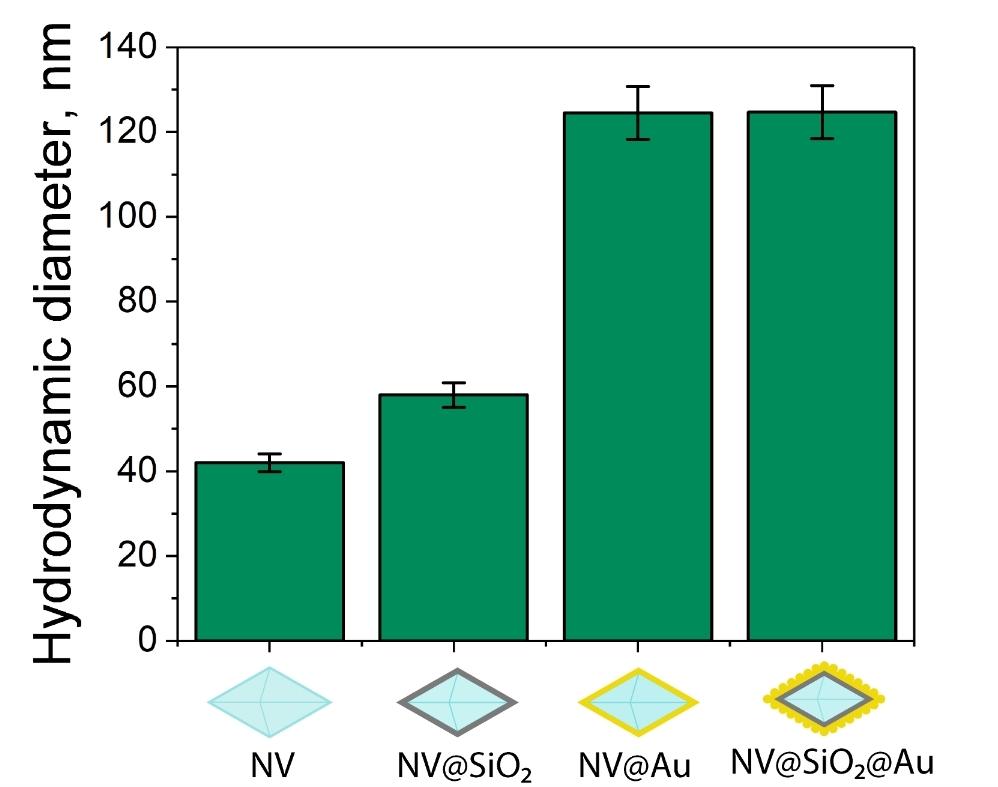


**Figure S2**. Hydrodynamic diameters of **NV**, **NV@SiO_2_**, **NV@Au**, **NV@SiO_2_@Au** derived from DLS measurements.

**Optical characterization**

- **Photoluminescence and dark-field measurements**

To perform photoluminescence measurements, a TrIOS setup was used, which provided three channels for optical spectroscopy measurements with top, side (oblique), and inverted (bottom) access. We used the bottom channel to pump the sample with the Torus solid state continuous wave 532 nm laser, and a Mitutoyo M Plan Apo VIS 50X NA 0.55 objective. We filtered the radiation at the output with a laser line 532 nm filter. The spectra were obtained using a Horiba LabRam HR spectrometer. The objective lens was used to focus the laser radiation on the sample.

We used the side channel for dark-field measurements. It included Avantes AvaLight-CAL-Neon-Mini lamp, optical fiber, collimator, and Mitutoyo M Plan Apo VIS 10X NA 0.28 objective located at an angle of about 65 degrees with respect to the normal of the sample.

The upper channel was used for signal acquisition. It included the Mitutoyo Plan Apo VIS 50X NA 0.55 objective, a camera and a spectrometer LabRAM HR. In the spectrometer, we used a 150 lines/mm grating and a detector Andor iDus 420. The optical scheme is shown in **Figure S3**.


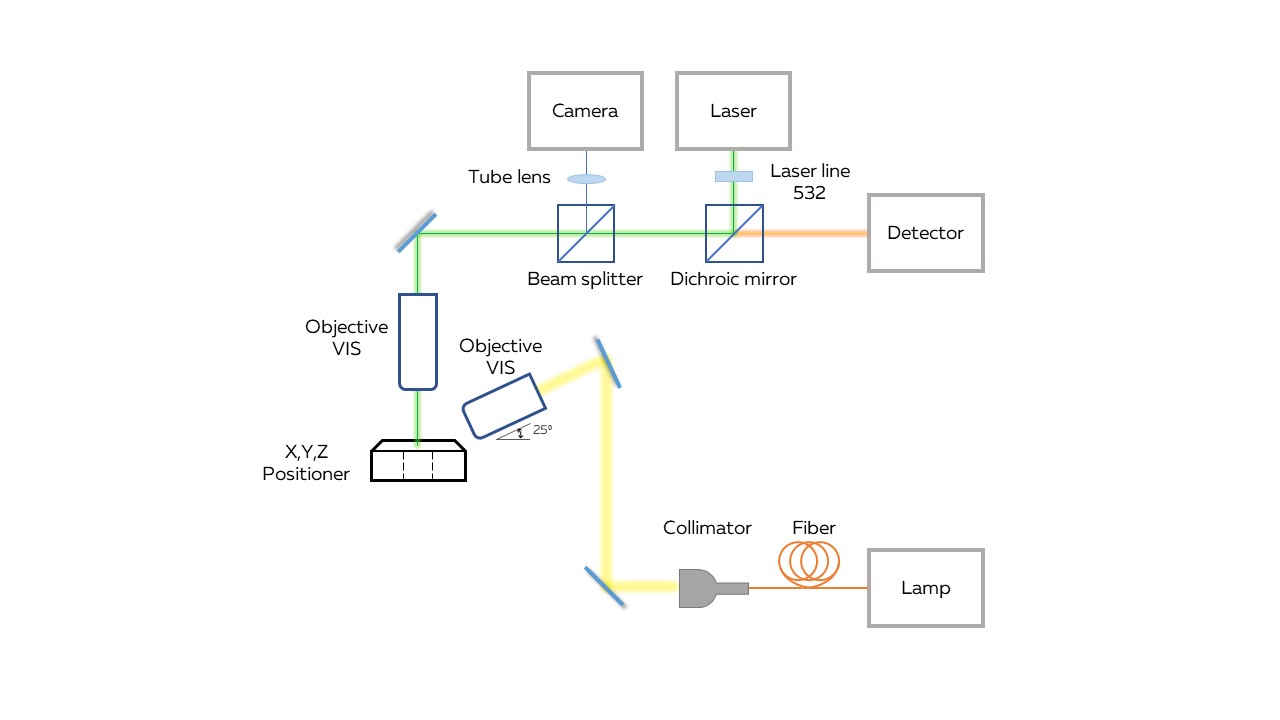
**Figure S3**. Optical scheme of photoluminescence and dark-field measurements.

- **Lifetime measurements**

To investigate the lifetime decay of our structures, we used a setup consisting of a laser pumping system, imaging system, and acquisition channel. The first part included a Picoquant PDL 800-D picosecond pulsed diode laser driver and a Picoquant LDH-FA 530XL head. The laser radiation passed through a Thorlabs DMLP-532 long-wavelength dichroic mirror and was focused by a Mitutoyo Plan Apo VIS 50X NA 0.42 objective. A 50:50 beam splitter and infinity-corrected tube lenses were applied to observe the sample image from the camera. A Notch 532 filter in the acquisition channel protected a single photon avalanche diode (SPAD) MPD PDM PD-100-CTC-FC. The acquisition signal was focused by an Olympus Plan N 10X NA 0.25 collimator into a multimode optical fiber connected to the SPAD. A TCSPC Picoquant Picoharp 300 module synchronized the signals from the laser driver and the SPAD. In the experiment, the laser frequency was equal to 80 MHz. The optical scheme is shown in **Figure S4**.


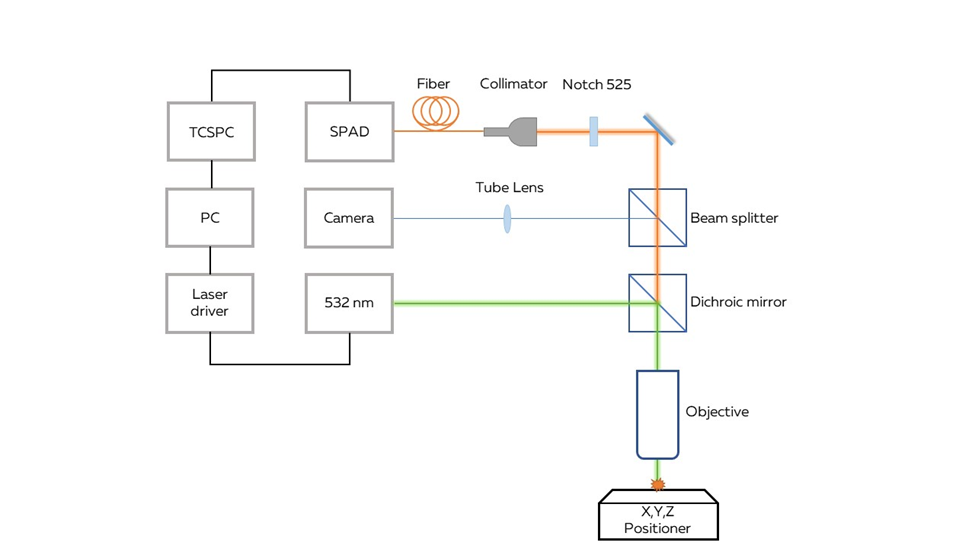


**Figure S4**. Optical scheme of lifetime measurements.

- **Optically detected magnetic resonance measurements**

The setup for ODMR measurement experiments can be roughly divided into three parts: laser channel, acquisition channel, and microwave part. The first part consists of a picosecond diode laser head Picoquant LDH-FA 530XL and a driver Picoquant PDL 800-D. The second part of the acquisition channel consists of a Mitutoyo Plan Apo VIS 50X NA 0.55 lens, camera, Notch 532 filter, collimator, fiber, single photon detector SPAD MPD PDM PD-100-CTC-FC, and time correlated single photon counting (TCSPC) module Picoquant Picoharp 300. The third part is the microwave part. It includes a Mini Circuit ZASWA-2-50DR+ microwave switcher, a microwave amplifier Agilent 83020A, a FPGA module National Instruments PCIe-7841, and a generator Rohde & Schwarz SMB-100A. The microwave resonant antenna was realized as a planar ring coil with a circular hole in the middle, inside of which resonant magnetic fields were concentrated. The parameters of the microwave resonant antenna were optimized to provide the resonance frequency at around 2.87 GHz and maximize the magnetic field inside the hole.^9^ The scheme is shown in **Figure S5**.


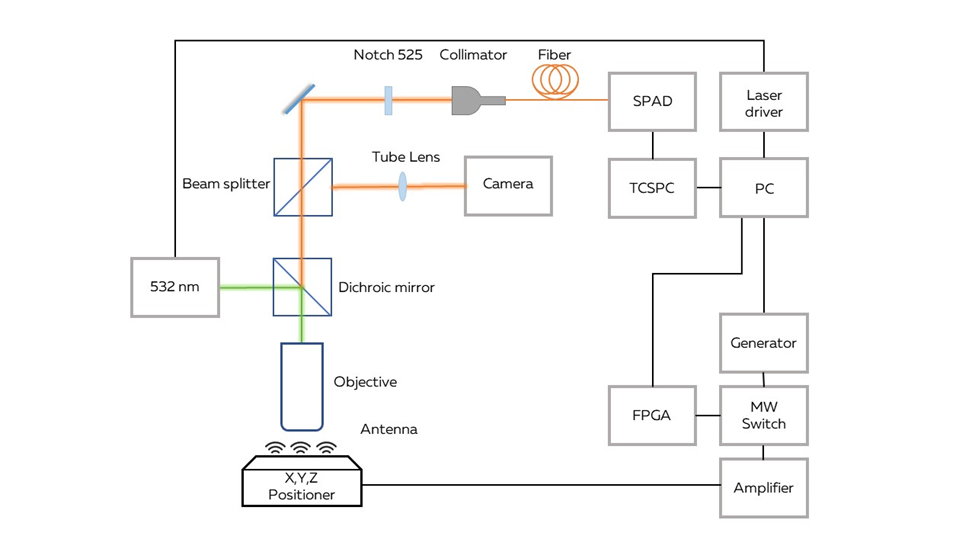


**Figure S5.** Optical scheme of ODMR measurements.

- **The operating principle of ODMR**

The investigated sample was placed on a sample holder connected with an XYZ positioner. The ODMR measurements were performed using a CW ODMR technique. The duration of each MW frequency was equal to 100 ms, the number of points was chosen as 100. The reference PL intensity was measured after each MW frequency. The laser was used in a quasi-continuous regime, when the repetition rate equals 80 MHz. Total time of complete measurements was 10 s. For that, the laser was turned on and continuously irradiated the sample. Meanwhile, the microwave key was switched on, and the microwave part of the setup started sweeping over different microwave frequencies with a collection of the luminescence intensity signal on the detector. Then, the microwave key was turned off, and the reference luminescence signal was saved and converted into the ODMR spectrum. The acquisition time was 1 ms for set microwave frequency. This process was repeated for each frequency. Then the laser power was changed, and the measurement procedure started over. The whole process is schematically demonstrated in **Figure S6**


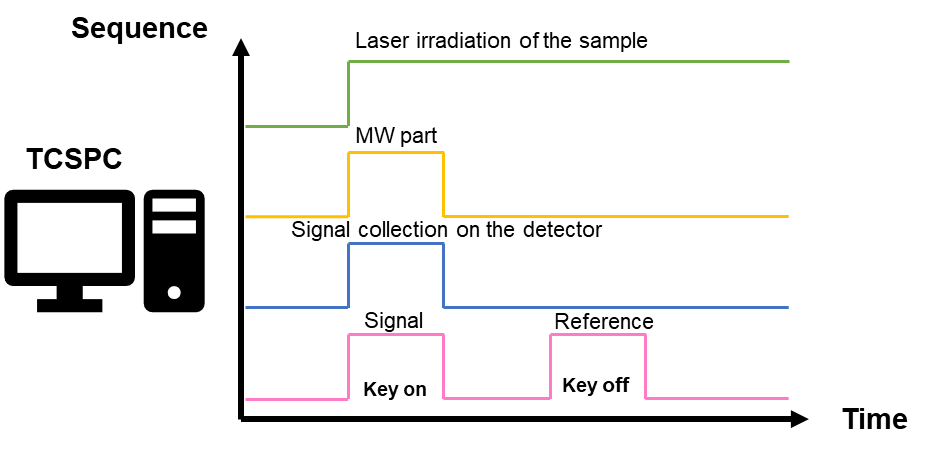
**Figure S6.** Working principle of ODMR.

- **Temperature determination and statistical analysis**

The resonance shift position of an ODMR peak can be linearly translated using the following equation:

$d\Delta/dT= -2\pi\times74.3 kHzK^{-1}$, (1)

where $\Delta$is the frequency of ODMR. The ODMR value for room temperature was taken as $2870 MHz.$ The examples of measured and fitted ODMR spectra without and with a biased magnetic field are shown in the **Figure S7**.

The temperature accuracy was estimated by standard deviation using the following equation:

$\Delta f=\sqrt{\frac{1}{n}\sum_{i=1}^{n} {(f_{i}-\underline{f})}^{2}}$, (2)

where n is the number of temperature measurements for the same power, $f_{i}$ is the experimental value of ODMR peak, and $\underline{f}$ is the mean value of all ODMR peaks for the same power. Then the obtained standard deviation for ODMR frequency was converted into temperature using the ratio $74.3 kHz K^{-1}.$


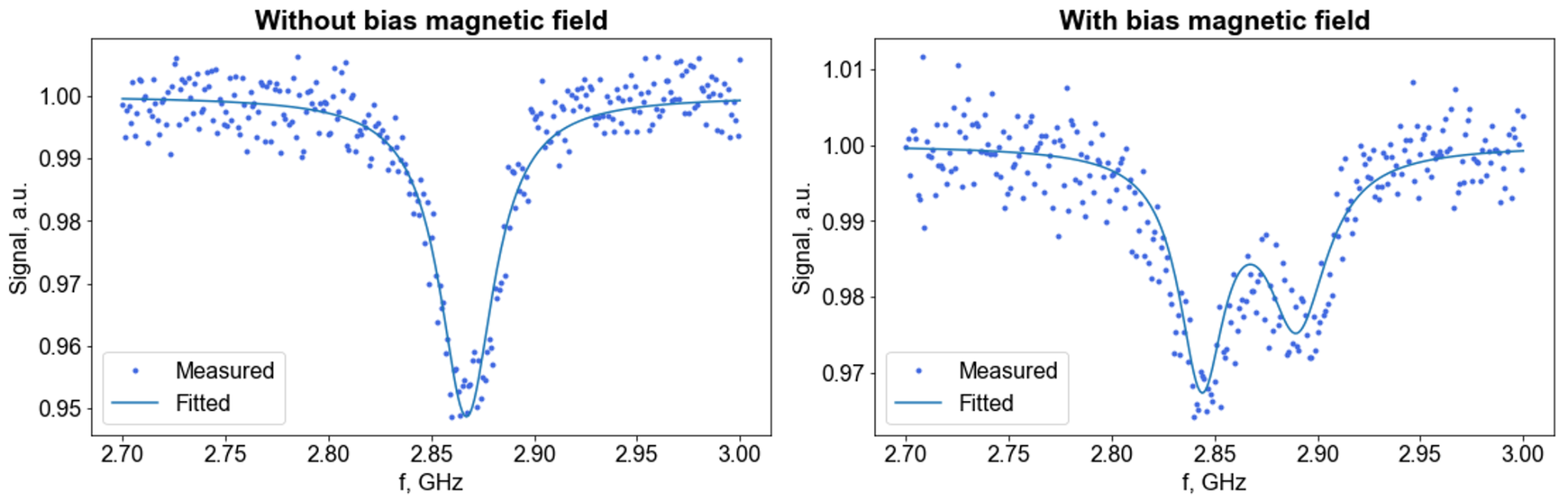


**Figure S7.** Representative measured and fitted ODMR spectra of **NV** at room temperature.

**Measurements of nanodiamond-based samples heating on glass**

To prepare samples for heating studies, 1μL of **NV**, **NV@SiO_2_**, **NV@Au,** and **NV@SiO_2_@Au** (1 μg/mL) were dropped on the glass and dried at room temperature. Afterwards, the sample was placed on a sample holder in the setup for ODMR measurements. Laser beam was focused on a single NP. To heat the NP, we varied the power density of the laser (0-6.57*10^6^ W/cm^2^). To determine the temperature, the ODMR peak position was recalculated into temperature. An example of the fitted ODMR spectra for different temperatures are given in **Figure S8**.


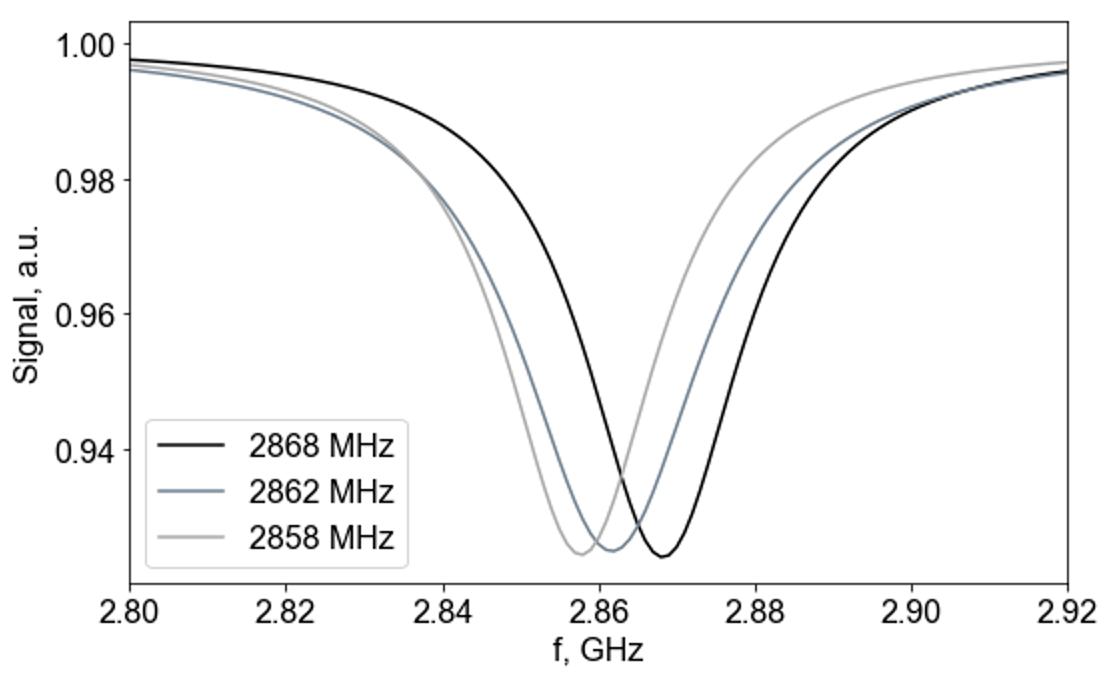


**Figure S8.** Representative fitted ODMR spectra of **NV@Au** at different temperatures.

**In vitro experiments**

- **Cells**

*Murine melanoma cell line (B16-F10 cells)* was obtained from the American Type Culture Collection. For cultivation of B16-F10 cells, AlphaMEM supplemented with 10% of vol. fetal bovine serum, 2 mM UltraGlutamine I and 1% of vol. antibiotic/antimycotic solution at 37°C was used. The culture was maintained in a sterile humidified atmosphere containing 95% air and 5% CO_2_.

- **Toxicity studies**

The toxicity of the **NV**, **NV@SiO_2_**, **NV@Au** and **NV@SiO_2_@Au** was estimated using the AlamarBlue assay. For this, B16-F10 cells were seeded into a 96-well plate (V = 200 µL, 100 000 cells/well). The next day, **NV**, **NV@SiO_2_**, **NV@Au** and **NV@SiO_2_@Au** were added to the cells at concentration 200 μg/mL. After 24 h, the cells were washed twice with PBS to remove non-internalized particles. Some wells with cells with internalized NPs were irradiated by a 532 nm laser with different power densities (5.2 W/cm^2^, 10.39 W/cm^2^, 15.59 W/cm^2^, 20.79 W/cm^2^, 30.66 W/cm^2^) for 1 min. Non-irradiated cells and cells without addition of NPs were used as controls. After irradiation, the old cell culture medium was replaced with a medium supplemented with 10% vol. of AlamarBlue and left for 4 h incubation. Then, cells were analyzed spectroscopically by measuring the absorption spectra at 570 and 600 nm with an UV−vis spectrophotometer (Thermo Scientific Multiskan GO). Measurements were repeated three times to obtain the mean value and the standard deviation.

- **Uptake studies**

To study the interaction of cells with nanodiamond-based samples, B16-F10 melanoma cells were placed in a confocal cell imaging dish (Eppendorf, diameter = 35 mm) at a density of 4.0*10^4^ cells per dish and left overnight. The following day, **NV@SiO_2_** labeled with Cy5 were introduced to the cells at concentration of 1 mg/mL. Next day, cells were washed with PBS and fixed with 4% paraformaldehyde. Additionally, Rhodamine B was used to stain the cell membranes. Cell imaging was performed with CLSM (Carl Zeiss LSM 710). Visualization of stained **NV@SiO_2_** labeled with Cy5 was performed with a 633 nm laser, while Rhodamine B membranes were excited by a 543 nm laser (emission filter 547 – 639 nm).

- **Temperature measurements inside cells**

To perform simultaneous temperature measurements and heating inside cells, B16-F10 cells were seeded into confocal cell imaging dishes (Eppendorf, d = 35 mm) at an amount of 5.0 x 10^4^ per dish and left overnight. The next day, **NV**, **NV@SiO_2_**, **NV@Au** and **NV@SiO_2_@Au** were added to the cells at concentration 200 μg/mL. Then, cells were washed twice with PBS and fixed with 1 mL of 4% paraformaldehyde for 1 h with the following washing step with PBS (1 mL). The solution with cells was dropped on a glass slide and covered with a second identical glass slide to prevent PBS evaporation. Afterwards, cells in-between two glass slides were placed on the sample holder, and ODMR spectra measurements were performed as described previously.

***Ex vivo* and *in vivo* studies**

- **Animals**

For in vivo studies, healthy C57BL/6 mice (males, 8-week-old, 18-22 g) were purchased from the St. Petersburg center of laboratory animals "Rappolovo" of the National Research Center "Kurchatov Institute" and were used for ex vivo and in vivo experiments, namely, ex vivo thermometry, biodistribution and therapy studies. Animals were maintained in accordance with the Guidelines for Accommodation and Care of Animals (European Convention for the Protection of Vertebrate Animals Used for Experimental and Other Scientific Purposes) and internal guidelines, and the experimental procedures were approved by the local authorities. Mice were housed in plastic cages at 22-24 °C and humidity of 40-60% under light/dark conditions for at least 14 d of quarantine, with free access to pellet food and water provided ad libitum, and were quarantined in a specific pathogen-free environment.

- **Tumor establishment**

In order to establish and grow the tumors in C57BL/6 mice (males, 8-week-old, 18-22 g), B16-F10 melanoma cells were used. For this, B16-F10 cells were harvested in the exponential growth phase by trypsinization. Then, the cells were washed twice with PBS and added in PBS solution at a concentration of 1x10^6^ cells/mL. Afterwards, the cells were subcutaneously injected (50 μL with concentration 1x10^6^ cells/mL) into the legs of C57BL/6 mice (0 d). Seven days after the tumor establishment (14 d after cell administration), the mice were inspected, and the animals were used for further experiments.

- **Cryogenic histological sample preparation**

To prepare samples for ex vivo temperature measurements, **NV**, **NV@SiO_2_**, **NV@Au** and **NV@SiO_2_@Au** (100 μL, 200 μg/mL) were injected into the tumor of mice. Next day, mice were euthanized by cervical dislocation, and their tumors were extracted.

For the sample preparation, extracted tumors injected with either **NV**, or **NV@SiO_2_**, or **NV@Au**, or **NV@SiO_2_@Au** were sliced using a cryogenic histological protocol to preserve fluids inside tissues. The thickness of slices was equal to 100 μm.

- **Ex vivo temperature measurements**

The prepared cryogenic histological samples injected with either **NV**, or **NV@SiO_2_**, or **NV@Au**, or **NV@SiO_2_@Au** were placed on the sample holder of the experimental setup and ODMR spectra were measured as described previously.

- **Biodistribution experiments**

Prior animal visualization, phantoms with different concentrations of Cy5-labeled **NV@SiO_2_** (500 𝜇g/mL, 250𝜇g/mL, 125𝜇g/mL, 62.50𝜇g/mL) were created and measured using IVIS Spectrum CT (Perkin Elmer) at 640/680 nm excitation/emission wavelengths (**Figure S9**). For biodistribution investigation, mice with tumors were divided into 3 groups: (i) hydratated mice, (ii) dehydrated mice, and (iii) hydrated mice with furosemide introduction. The first group had constant access to the water, whereas the second group remained dehydrated overnight. The third group also had constant access to the water, but 1 hour before the NPs’ administration, furosemide was injected into mice at a dose of 200 mg/kg, *per os*. The scheme of the experiment is shown in **Figure S10**. The scheme of the biodistribution experiments is described in **Figure S10.** Then, **NV@SiO_2_** labeled with Cy5 were intratumorally injected (100 μL, 500μg/mL). Animals were anesthetized with isoflurane and imaged using IVIS Spectrum CT (Perkin Elmer) at 640/680 nm excitation/emission wavelengths 1–24 h after the **NV@SiO_2_** injection. Average fluorescence intensities were measured in selected regions of interest (ROI) using Living Image 4.3 (Perkin Elmer).


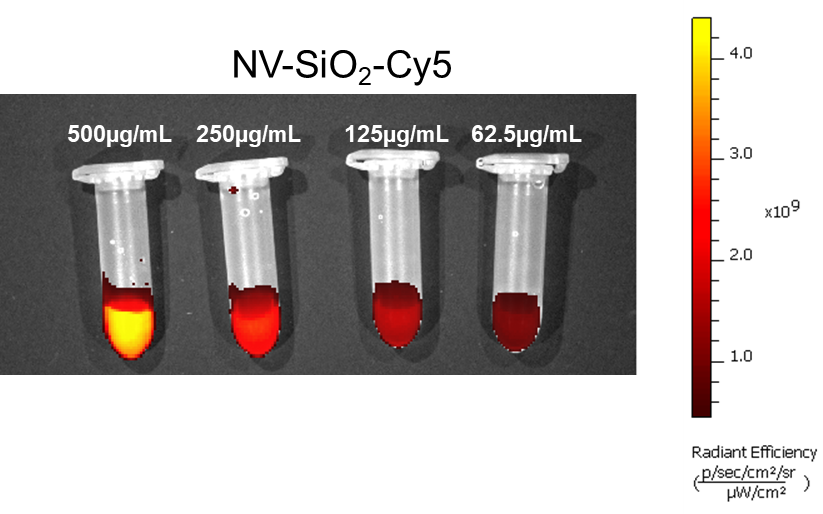


**Figure S9.** Fluorescence imaging scan of phantoms of different concentrations of NV@SiO_2_-Cy5.


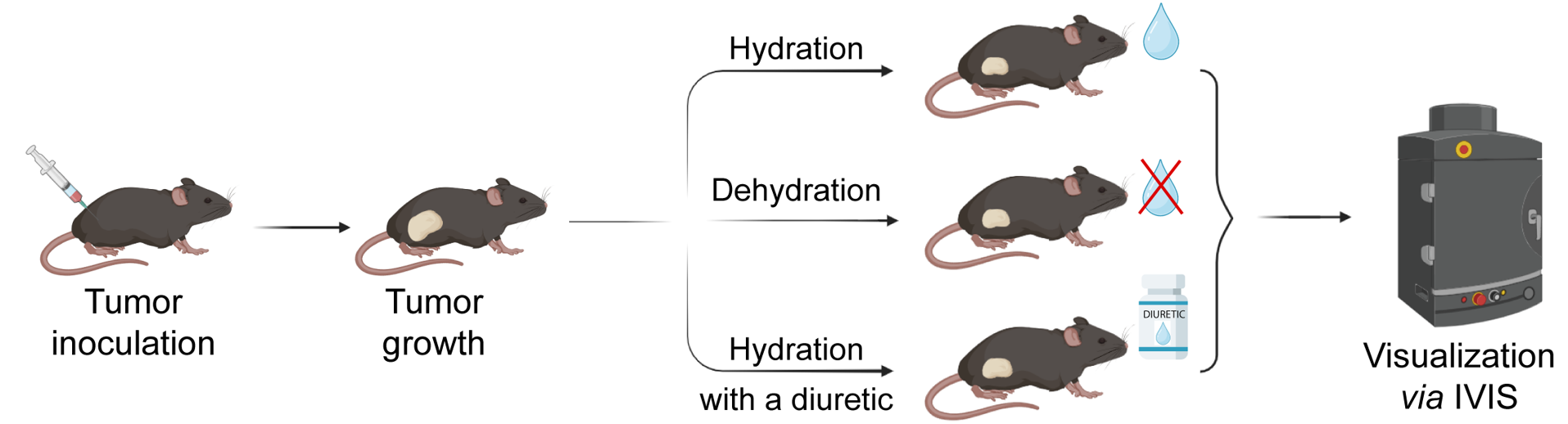


**Figure S10**. The plan of the biodistribution experiments

- **Photothermal therapy (PTT)**

To perform PTT, mice were divided into 4 groups: (i) with intratumoral injection of **NV@SiO_2_@Au** (100 𝜇g, 200 𝜇g/mL) and irradiation with laser, (ii) with intratumoral injection of **NV@SiO_2_@Au** without laser irradiation, (iii) only laser irradiation, and (iv) without laser irradiation. Prior to the laser treatment, the tumor area with and without injected **NV@SiO_2_@Au** was shaven to ensure uniform heating. For PTT, each mouse was located under a perpendicular directed laser beam of a continuous wave laser (Coherent Obis, 532 nm, 30.66 W/cm^2^). Before the irradiation, the animals were anesthetized with a mixture of zoletil and xyla in 0.9% NaCl. Mice were irradiated for 5 min and left for 24 h. The next day, mice were euthanized by cervical dislocation, and the therapy efficiency was examined by measuring the tumor volumes.


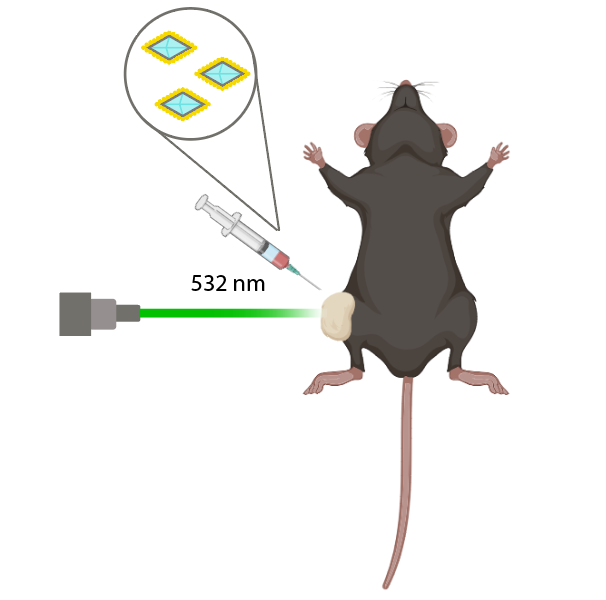


**Figure S11.** Optical scheme of photothermal therapy using **NV@SiO_2_@Au**.

- **Histological analysis**

For histological studies, mice were euthanized after PTT. Tumors were removed and preserved in a 10% formalin solution. Then, tumor tissues were fixed with 10% of formalin solution. Afterwards, the tissue samples were dehydrated and soaked in paraffin using isopropanol and Excelsior AS (Thermo, USA) device for histological processing. After transferring the samples into paraffin, using a rotary microtome HMS (Thermo, USA), 4 micrometer thick sections were sliced from paraffin blocks, placed on slides, stained with hematoxylin and eosin, and secured under cover glasses. Histological samples were examined in transmitted light using an Axio Imager microscope (Zeiss, Germany) and were scanned using a Pannoramic Midi (3D) micropreparation scanner (Histec, Hungary). The morphometric analysis was conducted using a camera AxioCam ICs 3 and AxioVision Rel programs. 4.8 (Zeiss, Germany).

**References**

1 A. Taylor, P. Ashcheulov, P. Hubík, Z. Weiss, L. Klimša, J. Kopeček, J. Hrabovsky, M. Veis, J. Lorinčík, I. Elantyev and V. Mortet, *Diam Relat Mater*, , DOI:10.1016/J.DIAMOND.2023.109837.

2 K. M. McPeak, S. V. Jayanti, S. J. P. Kress, S. Meyer, S. Iotti, A. Rossinelli and D. J. Norris, *ACS Photonics*, 2015, **2**, 326–333.

3 L. Gao, R. Lemarchand and M. Lequime, *Journal of the European Optical Society*, 2013, **8**, 13010.

4 S. Kedenburg, M. Vieweg, T. Gissibl and H. Giessen, *Opt Mater Express*, 2012, **2**, 1588.

5 D. J. Twitchen, C. S. J. Pickles, S. E. Coe, R. S. Sussmann and C. E. Hall, *Diam Relat Mater*, 2001, **10**, 731–735.

6 17.4: Heat Capacity and Specific Heat - Chemistry LibreTexts, https://chem.libretexts.org/Bookshelves/Introductory_Chemistry/Introductory_Chemistry_(CK-12)/17%3A_Thermochemistry/17.04%3A_Heat_Capacity_and_Specific_Heat, (accessed 8 May 2024).

7 Properties: Silica - Silicon Dioxide (SiO2), https://www.azom.com/properties.aspx?ArticleID=1114, (accessed 8 May 2024).

8 M. L. Huber, R. A. Perkins, D. G. Friend, J. V. Sengers, M. J. Assael, I. N. Metaxa, K. Miyagawa, R. Hellmann and E. Vogel, *J Phys Chem Ref Data*, , DOI:10.1063/1.4738955.

9 E. N. Gerasimova, V. V. Yaroshenko, P. M. Talianov, O. O. Peltek, M. A. Baranov, P. V. Kapitanova, D. A. Zuev, A. S. Timin and M. V. Zyuzin, *ACS Appl Mater Interfaces*, 2021, **13**, 36737–36746.

10 T. Zhang, G. Q. Liu, W. H. Leong, C. F. Liu, M. H. Kwok, T. Ngai, R. B. Liu and Q. Li, *Nat Commun*, , DOI:10.1038/s41467-018-05673-9.

11 L. Minati, C. L. Cheng, Y. C. Lin, J. Hees, G. Lewes-Malandrakis, C. E. Nebel, F. Benetti, C. Migliaresi and G. Speranza, *Diam Relat Mater*, 2015, **53**, 23–28.

12 R. Wang, X. Ji, Z. Huang, Y. Xue, D. Wang and W. Yang, *Journal of Physical Chemistry C*, 2016, **120**, 377–385.

13 E. N. Gerasimova, V. V. Yaroshenko, L. V. Mikhailova, D. M. Dolgintsev, A. S. Timin, M. V. Zyuzin and D. A. Zuev, *Adv Opt Mater*, 2022, **10**, 1–10.
